# Sensorimotor feedback loops are selectively sensitive to reward

**DOI:** 10.7554/eLife.81325

**Published:** 2023-01-13

**Authors:** Olivier Codol, Mehrdad Kashefi, Christopher J Forgaard, Joseph M Galea, J Andrew Pruszynski, Paul L Gribble

**Affiliations:** 1 Brain and Mind Institute, University of Western Ontario London Canada; 2 Department of Psychology, University of Western Ontario London Canada; 3 School of Psychology, University of Birmingham Birmingham United Kingdom; 4 Department of Physiology & Pharmacology, Schulich School of Medicine & Dentistry, University of Western Ontario Ontario Canada; 5 Robarts Research Institute, University of Western Ontario London Canada; 6 Haskins Laboratories New Haven United States; Peking University China; University of Oxford United Kingdom

**Keywords:** reinforcement learning, sensorimotor feedback, motor control, proprioception, vision, Human

## Abstract

Although it is well established that motivational factors such as earning more money for performing well improve motor performance, how the motor system implements this improvement remains unclear. For instance, feedback-based control, which uses sensory feedback from the body to correct for errors in movement, improves with greater reward. But feedback control encompasses many feedback loops with diverse characteristics such as the brain regions involved and their response time. Which specific loops drive these performance improvements with reward is unknown, even though their diversity makes it unlikely that they are contributing uniformly. We systematically tested the effect of reward on the latency (how long for a corrective response to arise?) and gain (how large is the corrective response?) of seven distinct sensorimotor feedback loops in humans. Only the fastest feedback loops were insensitive to reward, and the earliest reward-driven changes were consistently an increase in feedback gains, not a reduction in latency. Rather, a reduction of response latencies only tended to occur in slower feedback loops. These observations were similar across sensory modalities (vision and proprioception). Our results may have implications regarding feedback control performance in athletic coaching. For instance, coaching methodologies that rely on reinforcement or ‘reward shaping’ may need to specifically target aspects of movement that rely on reward-sensitive feedback responses.

## Introduction

If a cat pushes your hand while you are pouring a glass of water, a corrective response will occur that acts to minimize spillage. This simple action is an example of a behavioral response triggered by sensing a relevant change in the environment—here, a push that perturbs the movement of your arm away from the intended movement goal. This form of feedback control requires the brain to integrate sensory information from the periphery of the body, and thus suffers from transmission delays inherent to the nervous system. However, there is evidence that when more is at stake, we react faster to respond to demands of the task (Reddi and Carpenter, 2000). For instance, if wine was being poured instead of water, and your favorite tablecloth covers the table, you may be faster at correcting for a perturbation that risks spilling your wine.

**Figure 1.**
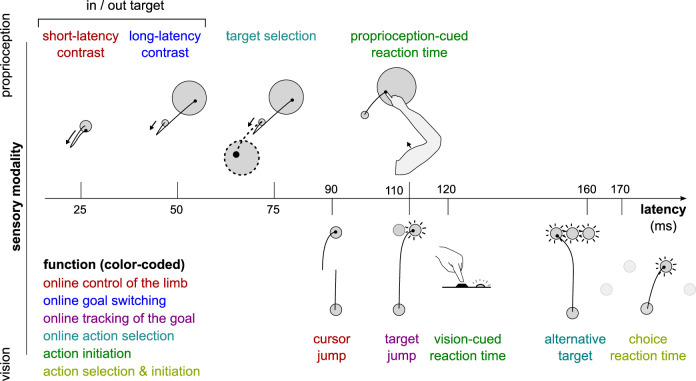
Different sensorimotor feedback responses are emphasized in different task designs. Feedback responses can be classified along three dimensions: the sensory modality on which they rely (vertical axis), their post-perturbation latency (horizontal axis), and the function they perform (color-coded). Latencies indicated here reflect the fastest reported values from the literature and not necessarily what was observed in this study. Note that this is a partial inventory. Figure 1 has been adapted from Figure 2 in Scott, 2016.

In the context of human motor control, feedback-based control is not a monolithic process (Reschechtko and Pruszynski, 2020; Scott, 2016). Rather, the term encompasses a series of sensorimotor feedback loops that rely on different sensory information, are constrained by different transmission delays (Figure 1), and are supported by different neural substrates (Reschechtko and Pruszynski, 2020). For instance, the circuitry underlying the short-latency rapid response (SLR) is entirely contained in the spinal cord (Liddell and Sherrington, 1924). The long-latency rapid response (LLR) relies on supraspinal regions such as the primary motor and primary sensory cortices (Cheney and Fetz, 1984; Day et al., 1991; Evarts and Tanji, 1976; Palmer and Ashby, 1992; Pruszynski et al., 2011), and is modulated by upstream associative cortical regions (Beckley et al., 1991; Graaf et al., 2009; Omrani et al., 2016; Takei et al., 2021; Zonnino et al., 2021). Visuomotor feedback responses rely on visual cortex and other cortical and subcortical brain regions (Day and Brown, 2001; Desmurget et al., 2004; Pruszynski et al., 2010). Due to these differences, each feedback response is sensitive to different objectives such as maintenance of a limb position or reaching toward a goal (Figure 1; Scott, 2016). Therefore, to address whether sensorimotor feedback control is sensitive to motivational factors requires testing multiple perturbation-induced feedback responses that rely on a distinct set of feedback loops. Here, the term ‘feedback response’ refers to a behavioral response to an externally applied perturbation. The term ‘feedback loop’ refers to a neuroanatomical circuit implementing a specific feedback control mechanism that will lead to all or part of a behavioral feedback response. In this work, we employed rewarding outcomes (specifically, monetary reward) as a means to manipulate motivation (Codol et al., 2020b; Galea et al., 2015; Goodman et al., 2014; Hübner and Schlösser, 2010; McDougle et al., 2021).

Recent work has demonstrated that rewarding outcomes improve motor performance in many ways. Reward results in changes to the speed-accuracy trade-off, a hallmark of skilled performance (Codol et al., 2020a; Codol et al., 2020b; Manohar et al., 2015; Manohar et al., 2019). Reward can lead to a reduction in noise in the central nervous system and at the effector to improve the control of movement (Codol et al., 2020a; Goard and Dan, 2009; Manohar et al., 2015; Pinto et al., 2013). But whether reward modulates sensorimotor feedback control specifically remains scarcely tested, although previous work in saccadic eye movements (Manohar et al., 2019) and indirect evidence in reaching (Codol et al., 2020b) suggests this may be the case. Some studies outline a general sensitivity of feedback control to reward during reaching but do not differentiate between each distinct feedback loop that the nervous system relies on to implement this control (Carroll et al., 2019; De Comité et al., 2022; Poscente et al., 2021). However, the information to which each loop is tuned greatly varies (Reschechtko and Pruszynski, 2020; Scott, 2016), and consequently it is unlikely that they are all uniformly impacted by reward.

In the present study, we tested how seven distinct sensorimotor feedback responses are modulated by reward. We measured feedback latency (how long does it take for a corrective response to arise) and feedback gain (how large is the corrective response) for each feedback response within rewarded and unrewarded conditions. Motivational factors can take different forms, such as rewarding or punishing outcomes (Chen et al., 2017; Chen et al., 2018a; Chen et al., 2018b; Codol et al., 2020b; Galea et al., 2015; Guitart-Masip et al., 2014), inhibition versus movement (Chen et al., 2018b; Guitart-Masip et al., 2014), contingency (Manohar et al., 2017), expectation (Lowet et al., 2020; Schultz et al., 1997), urgency (Poscente et al., 2021), or agency (Parvin et al., 2018). In this study, we focused on contingent rewarding outcomes, in which participants have agency over the returns they obtain, and with an expectation component since potential for returns is indicated at the start of each trial (see Results and Methods sections).

## Results

**Figure 2.**
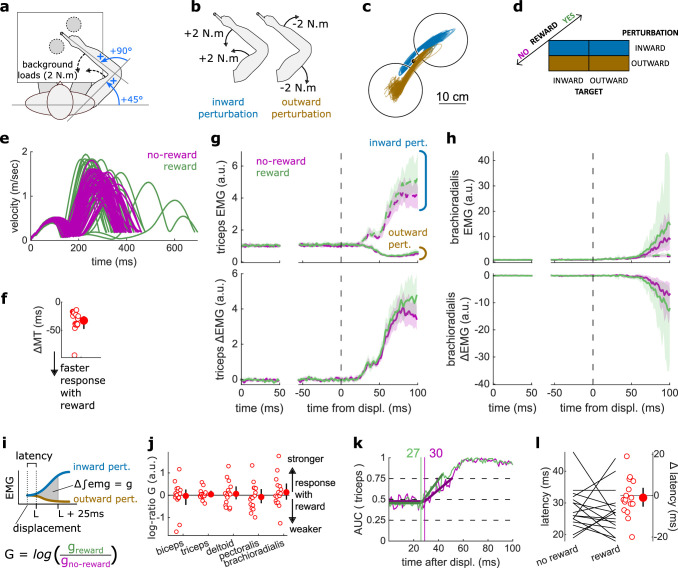
Results for the SLR contrast. (**a**) Schematic representation of the apparatus from a top view. Participants could move their arm in a horizontal plane. Background forces were applied to pre-activate the extensor muscles (dashed arrows). The dashed circles indicate the two possible target positions. (**b**) A mechanical perturbation, either two positive (inward) or two negative (outward) torques, was applied at the shoulder and elbow joints. To observe the SLR, we contrasted the feedback response in trials with inward torques against those with outward torques. (**c**) Example trajectories for one participant for inward (blue) and outward (brown) perturbations. Only trials with a target opposite to the perturbation are shown for clarity. (**d**) Schematic representation of the In-Out task’s full 2×2×2 factorial design, with the conditions color-coded as in (**b**). (**e**) Example participant’s radial hand velocity during trials with and without reward. (**f**) Difference in median movement time between rewarded and non-rewarded trials. (**g**) Mean triceps EMG signal across participants, with the dashed and solid lines representing inward and outward perturbations, respectively; bottom panels: difference between EMG signals following inward and outward perturbations. The left panels show EMG at trial baseline (see EMG signal processing). Shaded areas indicate 95% CIs. (**h**) Same as (**g**) for the brachioradialis. (**i**) Schematic of the method used to estimate feedback gains for the SLR. For each recorded muscle, the feedback gain g was defined as the difference between integrated EMG from the divergence point L between the contrasted conditions’ EMG signals to 25 ms post-divergence. We then computed a log-ratio G between the gain in rewarded and non-rewarded conditions. (**j**) Log-ratio G of feedback gains in the rewarded versus non-rewarded conditions in a 25 ms window following SLR onset. (**k**) Example area under the curve (AUC) to obtain response latency for one participant. Thick lines indicate line-of-best-fit for a two-step regression (see Materials and methods). (**l**) Response latencies. In all panels with a red filled dot and black error bars, the filled dot indicates the group mean and error bars indicate 95% CIs (*N*=16). CI, confidence interval; EMG, electromyographic; SLR, short-latency rapid response.

**Figure 3.**
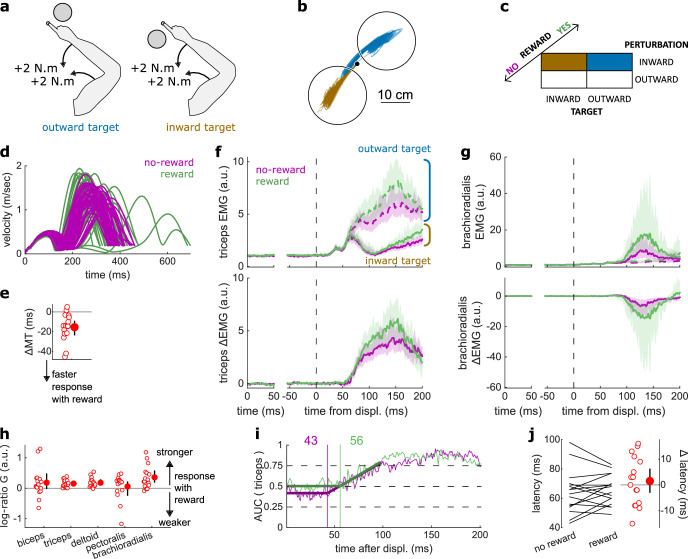
Results for the LLR contrast. (**a**) Contrast used to observe the LLR. Background loads are not drawn here for clarity. (**b**) Example trajectories for one participant for an outward (blue) or inward (brown) target. (**c**) Schematic representation of the In-Out task’s full 2×2×2 factorial design with the conditions color-coded as in (**b**). (**d**) Example participant’s radial hand velocity during trials with and without reward. (**e**) Difference in median movement time between rewarded and non-rewarded trials. (**f**) Mean triceps EMG signal across participants, with the dashed and solid lines representing the outward and inward target conditions, respectively, as indicated in (**a**); bottom panels: difference between the outward and inward target condition. The left panels show EMG at trial baseline (see EMG signal processing). Shaded areas indicate 95% CIs. (**g**) Same as (**f**) for the brachioradialis. (**h**) Log-ratio G of feedback gains in the rewarded versus non-rewarded conditions in a 50 ms window following LLR onset. (**i**) Example area under the curve (AUC) to obtain response latency for one participant. Thick lines indicate line-of-best-fit for a two-step regression (see Materials and methods). (**j**) Response latencies. In all panels with a red filled dot and black error bars, the filled dot indicates the group mean and error bars indicate 95% CIs (*N*=16). CI, confidence interval; EMG, electromyographic; LLR, long-latency rapid response.

We first assessed feedback gain and latency for the SLR and LLR, which are the fastest feedback responses observed in human limb motor control. The SLR corrects the limb position against mechanical perturbations regardless of task information, whereas the LLR integrates goal-dependent information into its correction following a mechanical perturbation (Pruszynski et al., 2014; Weiler et al., 2015; Weiler et al., 2016). Participants were seated in front of a robotic device that supported their arm against gravity and allowed for movement in a horizontal plane (Figure 2a). They positioned their index fingertip at a starting position while countering a +2 N·m background load (dashed arrows in Figure 2a) to activate the elbow and shoulder extensor muscles. We recorded electromyographic (EMG) signals using surface electrodes placed over the brachioradialis, triceps lateralis, pectoralis major (clavicular head), posterior deltoid, and biceps brachii (short head). After participants held their hand in the starting position for 150–200 ms, a 10 cm radius target appeared at 20° either inward (closer to the chest) or outward (away from the chest) with respect to the elbow joint. Next, a ±2 N·m torque perturbation was generated by the robot about the elbow and shoulder joints (Figure 2b). A positive or negative torque signifies an inward or an outward perturbation from the starting position, respectively. Participants were instructed to move their fingertip into the target as soon as possible after the perturbation occurred (Figure 2c). That is, the perturbation acted as a cue to quickly move the hand into the displayed target. This yielded a 2×2 factorial design (Figure 2d), in which an inward or outward perturbation was associated with an inward or outward target (Pruszynski et al., 2008). In the present study, we will refer to this task as the ‘In-Out Target’ task. Different contrasts allowed us to assess the SLR and LLR within this task (Figures 2b and 3a).

Unlike previous studies using this paradigm, we introduced monetary reward as a third factor to assess its impact on feedback responses (Figure 2d). Rewarded and non-rewarded trials were indicated at the beginning of each trial by displaying ‘$$$’ and ‘000’ symbols, respectively, on the display screen in front of participants. These symbols were replaced by each trial’s actual monetary value once the target was reached (always 0 ¢ in the case of non-rewarded trials). For rewarded trials, the monetary gains were proportional to the time spent inside the end target, therefore promoting faster reaches (see Materials and methods) because trial duration was fixed.

Feedback latency and gain were assessed by measuring when the EMG signal diverged between those two conditions, and the magnitude of the EMG signal following this divergence, respectively.

### The SLR remained similar in rewarded and non-rewarded conditions

The SLR can be assessed by contrasting the trials with an inward perturbation versus those with an outward perturbation (Figure 2b and d). Figure 2c shows trials falling in these categories for a typical participant. Note that the trials for which the target is in the direction of the perturbation were excluded from this visualization for clarity, but in practice they are included in the analyses and their inclusion or exclusion does not have a significant impact on the results. Before comparing the impact of rewarding context on feedback responses, we tested whether behavioral performance improved with reward by comparing movement times (MTs) expressed (see Statistical analysis for details). To do so, we compared each participant’s median MTs in the trials corresponding to the conditions of interest (Figure 2d) with a rewarding context to those with a non-rewarding context and compared them using a Wilcoxon rank-sum test. Indeed, median MTs were faster during rewarded trials than in non-rewarded ones (*W*=136, *r*=1, *p*=4.38e−4; Figure 2e–f).

The contrast successfully produced a clear divergence in EMG response between an inward and outward perturbation at the triceps (*triceps lateralis*, Figure 2g). This divergence is due to the geometry of the movement induced by the position of the targets and the direction of the background load (elbow flexion/extension against a background load that pre-loads the triceps). In comparison, other muscles such as the brachioradialis are not expected to diverge for the movements considered (Figure 2h). This highlights the methodological approach that we will consistently take across experiments: we design the geometrical layout of the two conditions we contrast to create a divergence of triceps EMG signal when the feedback response of interest arises, and take advantage of this divergence to compute a clear estimate of the feedback latency using a receiver operating characteristic (ROC) signal discrimination method (see Statistical analysis for details; Weiler et al., 2015). Because other muscles will not diverge, response latency cannot be assessed using these muscles (Figure 2h; Weiler et al., 2015).

Once latency is determined, we can compute the feedback gains for each of the five muscles in a time window following the response onset (i.e., the latency) for each participant. Specifically, feedback gains were defined as the difference between integrated EMG in the two contrasted conditions for each muscle during a 50 ms time window (Figure 2i; see Statistical analysis). Note that for the SLR only, we used a 25 ms time window to avoid overlap with the LLR response (Pruszynski et al., 2008). In the figures, we show the log-ratio G of these gains between rewarded and non-rewarded conditions, meaning a positive number indicates an increase in feedback gain for the rewarded conditions (Figure 2j).

For all muscles, we observed no difference in feedback gains following the onset of the SLR (biceps: *W*=78, *r*=0.57, *p*=0.61; triceps: *W*=81, *r*=0.6, *p*=0.5; deltoid: *W*=76, *r*=0.56, *p*=0.68; pectoralis: *W*=83, *r*=0.61, *p*=0.44; brachioradialis: *W*=80, *r*=0.59, *p*=0.53; Figure 2j). Next, we assessed the time of divergence of each participant’s EMG activity between inward and outward perturbation conditions using a ROC signal discrimination method (Figure 2k; Weiler et al., 2015). We performed this analysis on the rewarded and non-rewarded trials separately, yielding two latencies per participant. Latencies for the SLR in the non-rewarded conditions were in the 25 ms range post-perturbation, and latencies in the rewarded conditions were in a similar range as well, with no significant difference observed (*W*=70.5, *r*=0.52, *p*=0.57; Figure 2l). Therefore, rewarding outcomes affected neither feedback latency nor feedback gains of the SLR.

### Reward altered feedback responses as early as 50 ms post-perturbation

The LLR typically arises more strongly if the direction of a mechanical perturbation to the limb conflicts with the task goal (here, the target; Pruszynski et al., 2008). Therefore, turning to the LLR, we contrasted trials with an inward perturbation and an outward target with trials with an inward perturbation as well but an inward target instead (Figure 3a–c). We performed that contrast for non-rewarded trials, and then for rewarded trials independently. As a control, we compared each participant’s median MTs across both contrasts with a rewarding context to those with a non-rewarding context. We observed that MTs were shorter in rewarded trials (*W*=131, *r*=0.96, *p*=1.1e−3; Figure 3d–e).

Feedback gains were greater in the rewarded condition for the triceps, deltoid, and brachioradialis in a 50 ms window following LLR onset (biceps: *W*=98, *r*=0.72, *p*=0.12; triceps: *W*=136, *r*=1, *p*=4.4e−4; deltoid: *W*=135, *r*=0.99, *p*=5.3e−4; pectoralis: *W*=96, *r*=0.71, *p*=0.15; brachioradialis: *W*=129, *r*=0.95, *p*=1.6e−3; Figure 3f–h). Finally, ROC analysis showed that LLR latencies were similar in the rewarded condition compared to the non-rewarded condition (*W*=73, *r*=0.54, *p*=0.48; Figure 3i–j).

In summary, while the prospect of reward did not alter the SLR, it led to increases in feedback gains as early as the LLR, that is, about 50 ms post-perturbation, which is much earlier than the increase in latencies with reward reported in previous work (Carroll et al., 2019; De Comité et al., 2022).

### Latencies for selecting a target were reduced with reward

**Figure 4.**
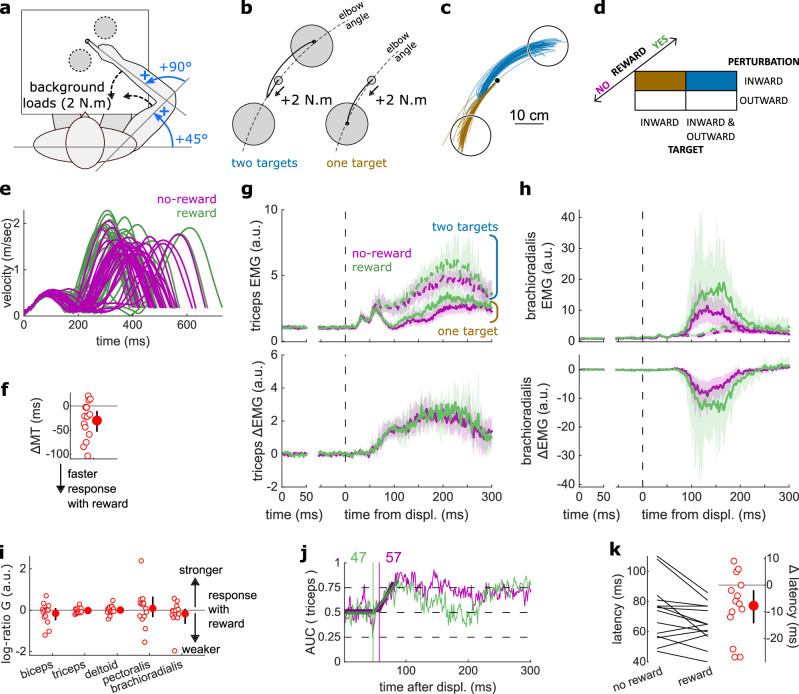
Results for the Target Selection task. (**a**) Schematic representation of the apparatus from a top view. Participants could move their arm in a horizontal plane. Background forces were applied to pre-activate the extensor muscles (dashed arrows). (**b**) Contrast used to observe the feedback response to a Target Selection. Background loads are not drawn for clarity. (**c**) Example trajectories for one participant in the two-targets (blue) and one-target (brown) conditions. (**d**) Schematic representation of the Target Selection task’s full 2×2×2 factorial design with the conditions color-coded as in (**b**). (**e**) Example participant’s radial hand velocity during trials with and without reward. (**f**) Difference in median movement time (MT) between rewarded and non-rewarded trials. A negative value indicates a smaller MT for rewarded trials. (**g**) Mean triceps EMG signal across participants, with the dashed and solid lines representing two- and one-target conditions, respectively, as indicated in (**b**); bottom panels: difference between the two- and one-target condition. The left panels show EMG at trial baseline (see EMG signal processing). Shaded areas indicate 95% CIs. (**h**) Same as (**g**) for the brachioradialis muscle. (**i**) Log-ratio *G* of feedback gains in the rewarded versus non-rewarded conditions in a 50 ms window following Target Selection response onset. (**j**) Example area under the curve (AUC) to obtain response latency for one participant. Thick lines indicate line-of-best-fit for a two-step regression (see Materials and methods). (**k**) Response latencies. In all panels with a red filled dot and black error bars, the filled dot indicates the group mean and error bars indicate 95% CIs (*N*=14). CI, confidence interval; EMG, electromyographic.

In addition to the SLR and LLR, slower feedback responses also exist that control for higher-level aspects of movement, such as selecting a target based on external cues (Figure 1). We tested the effect of reward on this feedback response in a ‘Target Selection’ task, which used the same apparatus and layout as the In-Out task (Figure 4a). In that task, participants were instructed to select a target based on the direction of a mechanical perturbation (Figure 4b). Specifically, half of the trials (112/224) contained two targets, and participants were instructed to reach to the target opposite to the perturbation direction following perturbation onset (Figure 4b, and blue trajectories in Figure 4c). In the other half of trials, only one target was displayed, and participants were instructed to reach to that target following perturbation onset, bypassing the need for any ‘selection’ process (brown trajectories). Therefore, the divergence point between each condition is the earliest behavioral evidence of the participant selecting a target and committing to it. For both one- and two-target conditions, outward and inward perturbations occurred equally to make the perturbation direction unpredictable (Figure 4d).

Similar to the previous experiments monitoring the SLR and LLR, participants were rewarded for shorter MTs. We computed for each participant the median MT of trials corresponding to the conditions of interest (Figure 4b–d) for rewarded and non-rewarded trials and compared them using a Wilcoxon rank-sum test. Performance improved in the rewarding condition (*W*=92, *r*=0.88, *p*=0.011; Figure 4e–f). This was not associated with an immediate increase in feedback gains (biceps: *W*=41, *r*=0.39, *p*=0.5; triceps: *W*=68, *r*=0.65, *p*=0.36; deltoid: *W*=54, *r*=0.51, *p*=0.95; pectoralis: *W*=59, *r*=0.56, *p*=0.71; brachioradialis: *W*=70, *r*=0.67, *p*=0.3; Figure 4g–i) but to a shortening of response latencies (*W*=76, *r*=0.72, *p*=0.031; Figure 4j–k).

### Proprioception-cued reaction times improved with reward

Reaction times have been measured in many different settings that include rewarding feedback (Douglas and Parry, 1983; Steverson et al., 2019; Stillings et al., 1968). The consensus is that reaction times are reduced when reward is available. However, previous work always considered reaction times triggered by non-proprioceptive cues, such as auditory (Douglas and Parry, 1983) or visual cues (Stillings et al., 1968). Here, we assessed participants’ reaction times triggered by a proprioceptive cue, which for arm movement tasks produce faster response latencies than visual cues (Pruszynski et al., 2008).

**Figure 5.**
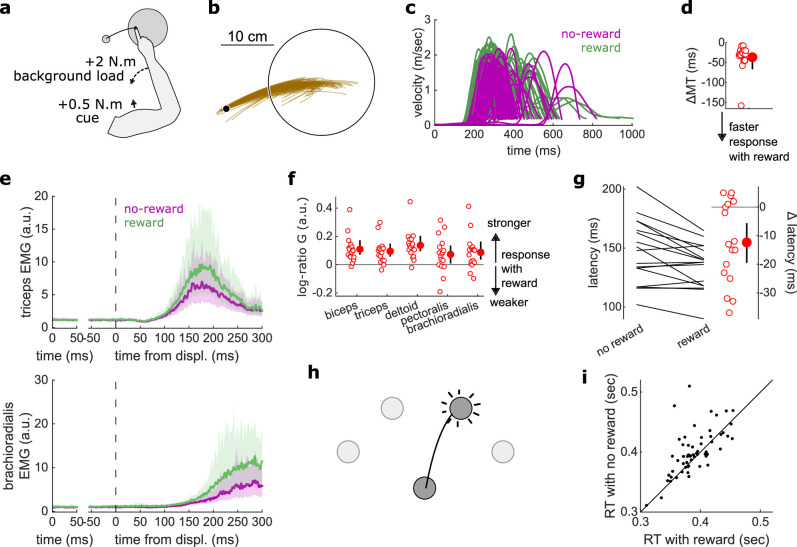
Results for the Reaction Time tasks. (**a**) Schematic of task design for Proprioception-cued Reaction Times. Participants were informed to initiate an elbow extension by a small mechanical perturbation at the shoulder (solid black arrow). Background loads pre-loaded the elbow extensor muscles (dashed black arrow). (**b**) Example trajectories for one participant. (**c**) Example participant’s radial hand velocity during trials with and without reward. (**d**) Difference in median movement time between rewarded and non-rewarded trials. (**e**) Top panels: mean triceps EMG signal across participants. The left panels show EMG at trial baseline (see EMG signal processing). Shaded areas indicate 95% CIs. Bottom panels: same as top panels for the brachioradialis. (**f**) Log-ratio of feedback gains in the rewarded versus non-rewarded conditions in a 50 ms window following the feedback response onset. (**g**) Response latencies. In all panels with a red filled dot and black error bars, the filled dot indicates the group mean and error bars indicate 95% CIs (*N*=17). (**h**) Schematic of task design for choice reaction times. (**i**) Median reaction times for each participant (*N*=60) in the choice reaction time task in the rewarded and non-rewarded conditions, plotted against the unity line. CI, confidence interval; EMG, electromyographic.

Participants held their arm so that the tip of the index finger was positioned at a starting location and the arm was stabilized against background loads that pre-loaded the forearm and upper arm extensor muscles (Figure 5a). A go cue was provided in the form of a small flexion perturbation at the shoulder, which led to less than 1° of shoulder or elbow rotation (Pruszynski et al., 2008). Participants were instructed to perform a fast elbow extension toward a target (10 cm radius) when they detected the go cue. While this experimental design is similar to the LLR contrast used in the In-Out Target experiment, a key distinction differentiates them. In the proprioception-cued reaction time task, the movement to perform can be anticipated and prepared for, while for the LLR the movement to perform depended on the direction of perturbation, which is unknown until the movement starts. Specifically, in the LLR, a perturbation toward or away from the target requires a stopping action to avoid overshooting or a counteraction to overcome the perturbation and reach the target, respectively. Therefore, the behaviour we are assessing in the reaction time task represents an initiation of a prepared action, rather than an online goal-dependent correction like the LLR.

Median MTs were greatly reduced in the rewarded condition compared to the non-rewarded condition (*W*=153, *r*=1, *p*=2.9e−4; Figure 5c–d), again indicating that the task successfully increased participants’ motivation. Reaction times were defined as when the (processed) triceps EMG signal rose 3 standard deviations above the trial baseline level (Pruszynski et al., 2008) for 5 ms in a row (Figure 5d). In line with the literature on reaction times triggered by other sensory modalities, proprioception-triggered reaction times were also reduced under reward, reducing on average by 12.4 ms, from 147.0 to 134.6 ms (*W*=117.5, *r*=0.77, *p*=0.01; Figure 5g). Feedback gains also increased significantly for all recorded muscles (biceps: *W*=151, *r*=0.99, *p*=4.2e−4; triceps: *W*=147, *r*=0.96, *p*=8.5e−4; deltoid: *W*=152, *r*=0.99, *p*=3.5e−4; pectoralis: *W*=123, *r*=0.8, *p*=0.028; brachioradialis: *W*=138, *r*=0.9, *p*=3.6e−3; Figure 5e–f).

Finally, we assessed reaction times in a choice reaction time task by re-analyzing a data set available online (Codol et al., 2020b). In this data set, participants (*N*=60) reached to one of four targets displayed in front of them in an arc centerd on the starting position (Figure 5h). Participants could obtain monetary reward for initiating their movements quicker once the target appeared (reaction times) and for reaching faster to the target (MTs). In line with the current study, reaction times were shorter in the rewarded than in the non-rewarded condition, from 400.8 to 390.2 ms on average (*W*=1241, *r*=0.67, *p*=0.016; Figure 5i). Of note, because EMG recordings were not available for the online data set, only kinematic data were available, which explains the slower absolute reaction times than reported in other studies (Haith et al., 2015; Summerside et al., 2018).

### Online visual control of limb position was unaltered by reward

**Figure 6.**
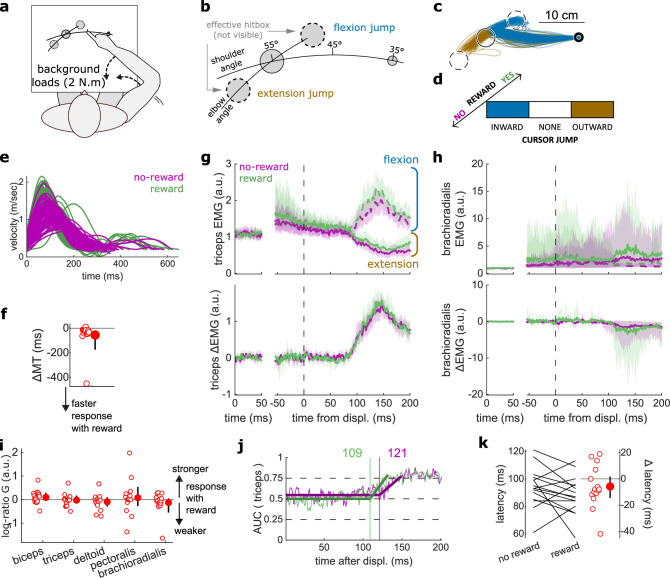
results for the Cursor Jump task. (**a**) Schematic representation of the apparatus from a top view. Participants could move their arm in a horizontal plane. 2 N·m Background forces were applied to pre-activate the extensor muscles (dashed arrows). (**b**) Contrast used to observe the feedback response to a cursor jump. (**c**) Example trajectories for one participant. The dashed circles correspond to where the actual target ‘hit box’ is to successfully compensate for the cursor jump. (**d**) Schematic representation of the Cursor Jump task’s full 2×3 factorial design with the conditions color-coded as in (**b**). (**e**) Example participant’s radial hand velocity during trials with and without reward. (**f**) Difference in median movement time between rewarded and non-rewarded trials. (**g**) Mean triceps EMG signal across participants, with the dashed and solid lines representing a flexion jump and an extension jump, respectively; bottom panels: difference between the flexion and extension condition. The left panels show EMG at trial baseline (see EMG signal processing). Shaded areas indicate 95% CIs. (**h**) Same as (**g**) but for the brachioradialis. (**i**) Log-ratio *G* of feedback gains in the rewarded versus non-rewarded conditions in a 50 ms window following the onset of the feedback response to the cursor jump. (**j**) Example area under the curve (AUC) to obtain response latency for one participant. Thick lines indicate line-of-best-fit for a two-step regression (see Materials and methods). (**k**) Response latencies. In all panels with a red filled dot and black error bars, the filled dot indicates the group mean and error bars indicate 95% CIs (*N*=15). CI, confidence interval; EMG, electromyographic.

Next, we assessed feedback response relying on visual cues rather than proprioceptive cues. In a new task using the same apparatus (Figure 6a), a visual jump of the cursor (indicating hand position) occurred halfway through the movement, that is, when the shoulder angle was at 45° like the Target Selection task (Figure 6b). This allowed us to assess the visuomotor corrective response to a change in perceived limb position (Dimitriou et al., 2013). To improve tuning of the triceps EMG signal to the feedback response, the reach and jump were specified in limb joint angle space, with the reach corresponding to a shoulder flexion rotation, and the cursor jump corresponding either to an elbow flexion or extension rotation (Figure 6a–c). A third of trials contained no jumps (Figure 6d). Like the experiments probing feedback responses relying on proprioception, participants were rewarded for shorter MTs.

Again, behavioral performance improved with reward, as measured by the difference in each participant’s median MT in the rewarded versus non-rewarded trials in the conditions of interest (Figure 6a; *W*=108, *r*=0.90, *p*=4.3e−3; Figure 6e–f), indicating that the rewarding context successfully increased participants’ motivation. Consistent with the previous experiments, we assessed feedback gains on all five recorded muscles in a time window of 50 ms following each participant’s response latency for the experiment considered (here a cursor jump). However, feedback gains did not immediately increase in the rewarded condition compared to the unrewarded condition (biceps: *W*=85, *r*=0.71, *p*=0.17; triceps: *W*=70, *r*=0.58, *p*=0.6; deltoid: *W*=76, *r*=0.63, *p*=0.39; pectoralis: *W*=73, *r*=0.61, *p*=0.49; brachioradialis: *W*=74, *r*=0.62, *p*=0.45; Figure 6g–i). Similarly, response latencies were not significantly different (*W*=71, *r*=0.59, *p*=0.26; Figure 6j–k).

### Feedback gains increased to respond to a visual Target Jump

**Figure 7.**
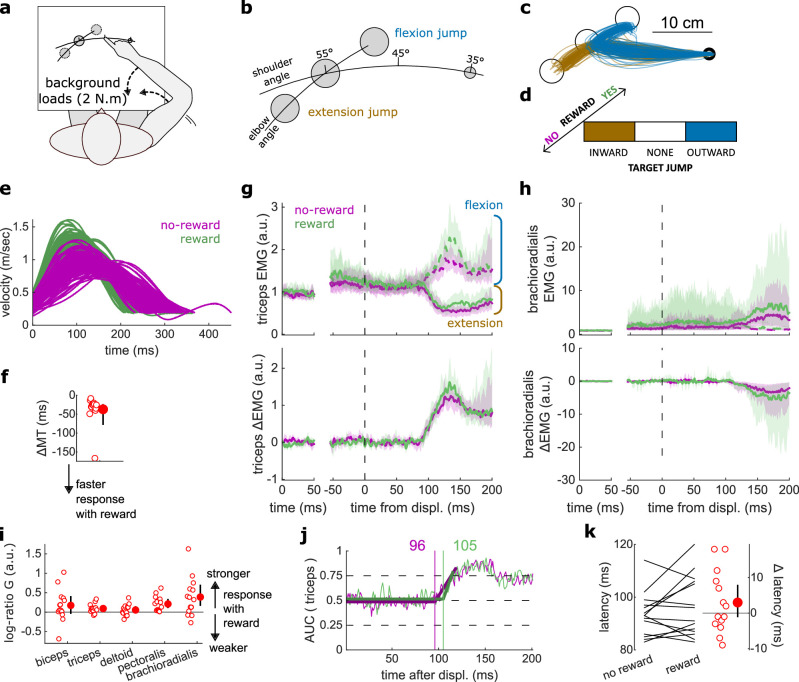
Results for the Target Jump task. (**a**) Schematic representation of the apparatus from a top view. Participants could move their arm in a horizontal plane. 2 N·m Background forces were applied to pre-activate the extensor muscles (dashed arrows). (**b**) Contrast used to observe the feedback response to a target jump. (**c**) Example trajectories for one participant. (**d**) Schematic representation of the Target Jump task’s full 2×3 factorial design with the conditions color-coded as in (**b**). (**e**) Example participant’s radial hand velocity during trials with and without reward. (**f**) Difference in median movement time between rewarded and non-rewarded trials. (**g**) Mean triceps EMG signal across participants, with the dashed and solid lines representing an extension jump and a flexion jump, respectively, as indicated in (**b**); bottom panels: difference between the extension and flexion conditions. The left panels show EMG at trial baseline (see EMG signal processing). Shaded areas indicate 95% CIs. (**h**) Same as (**g**) for the brachioradialis. (**i**) Log-ratio *G* of feedback gains in the rewarded versus non-rewarded conditions in a 50 ms window following the onset of the feedback response to the target jump. (**j**) Example area under the curve (AUC) to obtain response latency for one participant. Thick lines indicate line-of-best-fit for a two-step regression (see Materials and methods). (**k**) Response latencies. In all panels with a red filled dot and black error bars, the filled dot indicates the group mean and error bars indicate 95% CIs (*N*=14). CI, confidence interval; EMG, electromyographic.

Finally, we assessed the visuomotor feedback response arising from a visual shift in goal position using a Target Jump paradigm. The task design was identical to that of the Cursor Jump task (Figure 6a), except that the target, rather than the cursor, visually jumped in the elbow angle dimension (Figure 7a–d). Performance improved in the rewarding condition as well (*W*=103, *r*=0.98, *p*=3.7e−4; Figure 7e–f). Unlike for cursor jumps, feedback gains in the Target Jump task increased in the rewarding context for the triceps, pectoralis, and brachioradialis muscles (biceps: *W*=82, *r*=0.78, *p*=0.068; triceps: *W*=94, *r*=0.9, *p*=6.7e−3; deltoid: *W*=74, *r*=0.7, *p*=0.19; pectoralis: *W*=105, *r*=1, *p*=1.2e−4; brachioradialis: *W*=94, *r*=0.9, *p*=6.7e−3; Figure 7g–i). However, the response latencies remained similar between rewarded and non-rewarded conditions (*W*=67, *r*=0.64, *p*=0.39; Figure 7j–k).

## Discussion

**Figure 8.**
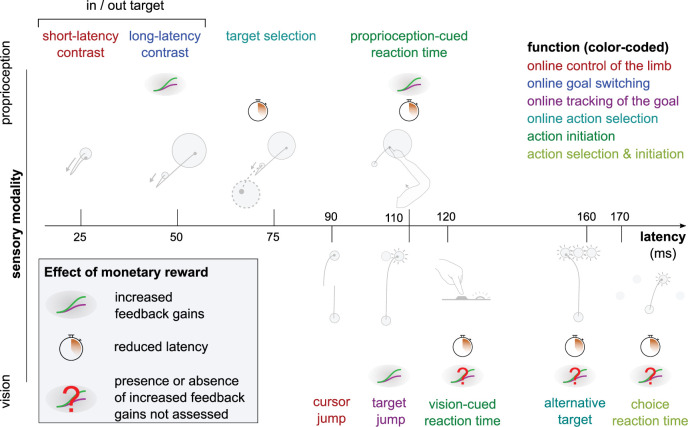
Overview of expected reward impact on sensorimotor feedback responses. Reward can impact a feedback loop response by increasing feedback gains or reducing latency. The color code indicates function and is identical to the one in Figure 1. Results for the Alternative Target and Vision-cued Reaction Time tasks are drawn from Carroll et al., 2019 and Stillings et al., 1968, respectively.

In this study, we tested whether reward affected seven different kinds of sensorimotor feedback responses through five experiments and re-analyzing results from an online data set (central column in Table 1). As expected, results indicate a heterogeneous sensitivity, both in terms of which feedback responses and which characteristics of the response were modulated by reward (Figure 8). The earliest effect was observed during the LLR response, that is, about 50 ms post-perturbation. This effect was constrained to the gain of the feedback response and did not extend to its latency. Following this, slower feedback responses in the proprioceptive domain were all affected by the prospect of reward (Figure 8). In the visual domain, the Target Jump task, and all slower feedback responses were affected as well. The fastest feedback responses for the proprioceptive and visual domain showed no modulation by reward, as shown by the SLR measurements and the visuomotor responses following cursor jumps, respectively.

**Table 1.** Task to feedback response mapping. This table indicates the correspondence between tasks and published work used, and the feedback responses assessed in the present study. RT, reaction time.

Feedback response	Task	Reference
SLR	In-Out Target task	
LLR		
Target Selection	Target Selection	
Target Jump	Target Jump	
Cursor Jump	Cursor Jump	
Proprioception-cued RTs	Proprioception-cued RTs	
Vision-cued RTs		Stillings et al., 1968
Alternative Target		Carroll et al., 2019
Choice RTs	(Data set re-analysed)	Codol et al., 2020a

### Shortening of response latencies may be constrained by transmission delays

The fastest feedback loops showed no reduction in latencies with reward, unlike the slower feedback loops, when adjusted for sensory modality (visual feedback loops tend to be slower than proprioceptive loops). The reproduction of this pattern both in vision and proprioception hints at a mechanism that occurs across sensory domains. Likely, the usual latencies of the fastest feedback loops are constrained by transmission delays. For instance, electrophysiological recordings in monkeys show that proprioceptive information for the LLR takes about 20 ms to reach the primary sensory cortex, and a response traveling back downward would take an additional 20 ms, explaining most of the ~50 ms latencies generally observed for this feedback loop (Cheney and Fetz, 1984; Omrani et al., 2016; Pruszynski et al., 2011). Consequently, the LLR has little room for latency improvements beyond transmission delays. This is well illustrated in the Proprioception-cued Reaction Time task, which holds similarities with the task used to quantify the LLR but with a smaller mechanical perturbation. Despite this similarity, latencies were reduced in the Proprioception-cued Reaction Time task, possibly because the physiological lower limit of transmission delays is much below typical reaction times.

As we move toward slower feedback loops, additional information processing steps take place centrally (Carroll et al., 2019; Nashed et al., 2014), which contributes to overall latencies. For instance, the Target Selection and Reaction Time tasks require accumulation of sensorimotor and/or cognitive evidence for selecting an action or triggering movement initiation, respectively. The time course of these processes can vary depending on urgency, utility, and value of information (Fernandez-Ruiz et al., 2011; Reddi and Carpenter, 2000; Steverson et al., 2019; Thorpe and Fabre-Thorpe, 2001; Wong et al., 2017). Therefore, we propose that a rewarding context leads to a reduction in latencies only for the feedback loops relying on online accumulation of sensorimotor and cognitive information, which benefit from that rewarding context. Conversely, typical latencies of the faster feedback loops cannot be shortened because transmission delays cannot be reduced below certain physiological values, regardless of the presence or absence of reward.

### Increase in feedback gains through anticipatory pre-modulation

Unlike transmission delays, the strength of feedback responses can be modulated before movement occurrence, that is, during motor planning (Graaf et al., 2009; Selen et al., 2012), which we will refer to as anticipatory pre-modulation here. For instance, the gain of the LLR response can vary due to anticipatory cognitive processes such as probabilistic information (Beckley et al., 1991) and verbal instructions (Hammond, 1956) provided before the start of a trial. We propose that this capacity to pre-modulate feedback gains may enable the distinct pattern of reward-driven improvements compared to latency shortenings (Figure 8).

Pre-modulation results from preparatory activity, which at the neural level is a change in neural activity prior to movement that will impact the upcoming movement but without producing overt motor activity during the pre-movement period—that is, output-null neural activity (Churchland et al., 2006; Elsayed et al., 2016; Vyas et al., 2020). Regarding feedback gain pre-modulation, this means that in the region(s) involved there is an output-null neural activity subspace from which the neural trajectory unfolding from the upcoming movement will respond differently to a perturbation. Importantly, not all preparatory activity will yield a modulation of feedback gain, or even task-dependent modulation at all. An extreme example of this distinction is the spinal circuitry, where preparatory activity is observed but does not necessarily translate into task-dependent modulation (Prut and Fetz, 1999). This is also consistent with our result, as we observe no change in feedback gain with reward in the SLR.

A corollary to our proposal is that feedback loops that do not show increased feedback gains with reward would also more generally not be susceptible to gain pre-modulation like that which occurs in the LLR (Beckley et al., 1991; Graaf et al., 2009; Takei et al., 2021; Zonnino et al., 2021). A task in our study that is suited to test this possibility is the Cursor Jump task, because it does not show feedback gain modulation, while the Target Jump task, which has a very similar design, does. Therefore, one could consider a probabilistic version of these tasks in which the probability of a jump in each direction is manipulated on a trial-by-trial basis, and participants are informed before each trial of the associated probability (Beckley et al., 1991; Selen et al., 2012). Previous work shows that this manipulation successfully modulates the LLR feedback gain of the upcoming trial (Beckley et al., 1991). Given our hypothesis, it should pre-modulate the feedback gain following a target jump, but not following a cursor jump, because the absence of reward-driven feedback gain modulation would indicate the circuitry involved is not susceptible to anticipatory pre-modulation.

To our knowledge, there is no evidence that a neuroanatomical region’s capacity for pre-modulation of feedback gains will depend on the transmission delay of its somatosensory afferents. Rather, this capacity is likely dependent on the nature of the information made available locally by bottom-up and/or top-down input drive (Remington et al., 2018; Seki and Fetz, 2012). Therefore, an important consequence of our proposal above is that there should be no central compensation mechanism taking place, that is, we should not expect feedback gains of a feedback loop to increase because latencies cannot be reduced. Feedback gains increasing before latencies are reduced may simply occur because it is easier, when possible, to have faster responses via pre-modulation, because it is done before the perturbation occurrence in the first place.

We hypothesize above that improved capacity to accumulate sensorimotor and cognitive evidence may underpin reduction in latencies for the feedback loops that rely on such process. It is unclear whether this improvement is due to improved processing of information online, or if it also stems from pre-modulation even before the accumulation of evidence starts (e.g., Sohn et al., 2019). In the latter case, a possibility is that changes in feedback gains and reduction in latency co-occur due to the similar nature of the pre-modulation process involved.

### A behavioral classification based on sensory domain and response latency

Several categorization schemes have been used in the literature to sort the large variety of feedback loops involved in feedback control: by typical response latency, by function, or by sensory modality (Figure 1; Forgaard et al., 2021; Pruszynski and Scott, 2012; Reschechtko and Pruszynski, 2020; Scott, 2016). From our results, categorizing feedback loops by typical latency range appears to be sensible, at least in the context of our observations, and potentially as a general principle. Unsurprisingly, categorization by sensory modality is relevant as well, not only because latency range is impacted by sensory modality, but also because the pathways, neural regions, and mechanisms involved are fundamentally distinct across modalities.

This leaves categorization by function (color-coded legend box in Figure 1) outstanding, as it does not match any observed pattern here (Figure 8). This is surprising, as categorization by function is a behavioral classification, and so one may expect it to yield greater explanatory power to interpret results of a behavioral, descriptive set of experiments like we report here. Therefore, while it may have value at a higher-order level of interpretation, our results suggest that a categorization of feedback loops based on function may not always be the most appropriate means of characterizing feedback control loops. This may partially stem from the inherent arbitrariness of defining function and assigning a specific task to that function. In contrast, categorization based on neural pathways, neural regions involved, and sensory modality may result in more insightful interpretations, because they are biologically grounded, and therefore objective means of categorization. More generally, our results provide additional evidence in favor of a bottom-up approach to understanding sensorimotor feedback control as opposed to a top-down approach based on a functional taxonomy. This approach is described as early as Sherrington (Burke, 2007; Sherrington, 1906), who put forward an organizational principle of the central nervous system tied to sensory receptor properties (extero-, proprio-, intero-ceptors, and distance-receptors). More recently, Loeb, 2012 proposed that the existence of an optimal high-order, engineering-like control design in the central nervous system is unlikely due to the constraints of biological organisms, a theory further detailed by Cisek, 2019 from an evolutionary perspective.

### Implications for optimal feedback control

The optimal feedback control (OFC) framework is a commonly used theoretical framework for explaining how movement is controlled centrally and producing behavioral predictions based on its core principles (Miyashita, 2016; Scott, 2012). One such principle is that feedback gains may be tuned according to a cost function to produce optimal control (Todorov, 2004). The results we report here generally agree with this principle, because we do observe modulation of feedback gains as we manipulate expectation of reward (and so expected cost of movement). However, to our knowledge, previous OFC implementations do not distinguish between individual feedback loops in that regard, that is, it is assumed that any feedback loop can adjust their gains to optimize the cost function (Scott, 2012; Shadmehr and Krakauer, 2008; Todorov, 2005). Our results suggest that not all feedback loops respond in this way, and that the OFC framework’s explanatory power may benefit from distinguishing between the loops that adjust their gains with reward, and those which do not.

### General considerations

While SLR circuitry is contained within the spinal cord, it receives supraspinal modulation and displays functional sensitivity to higher-order task goals (Crone and Nielsen, 1994; Nielsen and Kagamihara, 1992; Nielsen and Kagamihara, 1993; Prut and Fetz, 1999; Weiler et al., 2019; Weiler et al., 2021). Spinal reflex responses can also be modulated over weeks in the context of motor learning using operant conditioning (Wolpaw, 1987; Wolpaw and Herchenroder, 1990). Therefore, while unlikely, central modulation of SLR circuitry for rewarding outcomes during motor control could not be a priori ruled out. However, we observed no such modulation in the present experiments.

A feedback loop that we did not assess is the cortico-cerebellar feedback loop (Becker and Person, 2019; Chen-Harris et al., 2008; Manohar et al., 2019). This loop contributes to saccadic eye movements (Chen-Harris et al., 2008), which show performance improvements under reward as well (Manohar et al., 2015; Manohar et al., 2019). Electrophysiological evidence in mice (Becker and Person, 2019) and non-invasive manipulation in humans (Miall et al., 2007) suggest this loop also contributes to reaching movement, but behavioral assessment remains challenging.

### Conclusion

Our results combined with previous work (Carroll et al., 2019; Codol et al., 2020b; Douglas and Parry, 1983; Stillings et al., 1968) show that sensitivity to reward is not uniform across all feedback loops involved in motor control. Based on our observations, we propose that (1) reduction of latencies with reward is mainly dictated by neural transmission delays and the involvement (or lack) of central processes in the loop considered, and (2) increase of feedback gains with reward may be the result of central pre-modulation. We also argue against a ‘top-down’ classification of feedback loops based on function, and in favor of a ‘bottom-up’ classification based on neural pathways and regions involved. Finally, we propose potential refinements to apply to the OFC framework based on our results.

Together with previous work showing reduction in peripheral noise with reward (Codol et al., 2020b; Manohar et al., 2019), the results presented here enable us to further complete the picture on how rewarding information triggers improvements in motor performance at the behavioral level. Outstanding questions remain on how reward leads to improvements in motor control, such as whether noise reduction may also occur centrally (Goard and Dan, 2009; Manohar et al., 2015; Pinto et al., 2013), or whether the cortico-cerebellar feedback loop is also involved in reward-driven improvements (Becker and Person, 2019; Codol et al., 2020b; Miall et al., 2007). Beyond motor control, it remains to be tested whether the motor control improvements we observe could be assimilated through motor learning to systematically enhance athletic coaching (Hamel et al., 2019) and rehabilitation procedures (Goodman et al., 2014; Quattrocchi et al., 2017).

## Methods

### Data set and analysis code availability

All behavioral data and analysis code are freely available online on the Open Science Framework website at https://osf.io/7t8yj/. The data used for the Choice Reaction Time tasks are also available with the original study’s data set at https://osf.io/7as8g/.

### Participants

In total, 16, 15, 14, 14, and 17 participants took part in the In-Out target, Cursor Jump, Target Jump, Target Selection, and Proprioception-cued Reaction Time task, respectively, and were remunerated CA$12 or 1 research credit per hour, plus performance-based remuneration. To be eligible to participate, participants had to be between 18 and 50 years old, be right-handed, have normal or corrected-to-normal vision, and have no neurological or musculoskeletal disorder. Participants made on average 2.14, 2.83, 2.39, 3.80, and 3.19 Canadian cents per rewarded trial on the In-Out Target, Cursor Jump, Target Jump, Target Selection, and Proprioception-cued Reaction Time task, respectively, and earned on average in total $7.19, $4.24, $3.59, $4.26, and $1.72 from performance, respectively. All participants signed a consent form prior to the experimental session. Recruitment and data collection were done in accordance with the requirements of the Health and Sciences Research Ethics Board at Western University (ethics approval #115787).

### Apparatus

A BKIN Technologies (Kingston, ON) exoskeleton KINARM robot was used for all the tasks presented here. In all cases, the participant was seated in front of a horizontally placed mirror that blocked vision of the participant’s arm and reflected a screen above so that visual stimuli appeared in the same plane as the arm. EMG activity of brachioradialis, triceps lateralis, pectoralis major, posterior deltoid, and biceps brachii was recorded using wired surface electrodes (Bagnoli, Delsys, Natick, MA). EMG and kinematic data were recorded at 1000 Hz.

The participant’s arm was rested on a boom that supported the limb against gravity and allowed for movement in a horizontal plane intersecting the center of the participant’s shoulder joint. Pilot tests using an accelerometer fixed on the distal KINARM boom showed that logged perturbation timestamps corresponding to the onset of commanded robot torque preceded the acceleration of the distal end of the robot linkage by 4 ms. Perturbation timestamps were adjusted accordingly for the analysis of experimental data. For the visual feedback tasks (Cursor and Target Jumps), perturbation onsets were determined using a photodiode attached to the display screen (see *Target Jump and Cursor Jump Tasks* description in ‘Experimental design’ section below for details).

### Experimental design

#### General points

Background loads were used to pre-load extensor muscles to improve measurement sensitivity. In all tasks using mechanical perturbations, perturbation magnitudes were added to background loads. For instance, if a background torque load of –2 N·m was applied and a –4 N·m perturbation was specified, then during the perturbation the robot produced a –6 N·m torque.

In all tasks, the start position and the target(s) were the same color, which was either pink or cyan blue depending on whether the trial was rewarded or non-rewarded. Target color assignment to reward conditions was counterbalanced across participants.

Target sizes are in cm rather than in degrees because the degree measurements used here are with respect to joint angles. Therefore, a target with same angular size would result in different metric sizes for individuals with longer upper arm or forearm, due to angular projection (Figure 2a).

#### In-Out Target task

The location of the tip of the participant’s right index finger was indicated by a 3 mm radius white cursor. At the beginning of each trial, a 3 mm radius start position appeared, along with a reward sign below the target showing ‘000’ or ‘$$$’ to indicate a non-rewarded or a rewarded trial, respectively. The start position was located so that the participant’s external shoulder angle was 45° relative to the left-right axis (parallel to their torso), and the external elbow angle was 90° relative to the upper arm (Figure 2a). When participants moved the cursor inside the start position the cursor disappeared. It reappeared if the participant exited the start position before the perturbation onset. After the cursor remained inside the start position for 150–200 ms, a background torque (+2 N·m) ramped up linearly in 500 ms at the shoulder and elbow to activate the extensor muscles. Then, following another 150–200 ms delay, a 10 cm radius target appeared either at +20 or –20° from the start position (rotated about the elbow joint). Following target appearance and after a 600 ms delay during which we assessed baseline EMG activity for that trial (referred to as ‘trial baseline’ throughout the text), the robot applied a ±2 N·m perturbation torque at the elbow and shoulder joints (Figure 2a–c). This combination of load on the shoulder and elbow was chosen to create pure elbow motion, as the robot torque applied at the shoulder counteracted the interaction torque arising at the shoulder due to elbow rotation (Maeda et al., 2018; Maeda et al., 2020). Because the time interval between the onset of the visual target and the onset of the perturbation was fixed, we tested for anticipatory triceps EMG activity in a 20 ms window immediately before the perturbation onset. We observed no difference, both comparing the inward and outward perturbation conditions for the SLR (no reward: *W*=83, *r*=0.61, *p*=0.43; with reward: *W*=88, *r*=0.65, *p*=0.30) and comparing the inward and outward target conditions for the LLR (no reward: *W*=70, *r*=0.51, *p*=0.92; with reward: *W*=82, *r*=0.60, *p*=0.46). Following the mechanical perturbation, participants were instructed to move the cursor as fast as possible to the target and stay inside it until the end of the trial. Each trial ended 800 ms after perturbation onset, at which point the target turned dark blue, the reward sign was extinguished, and the final value of the monetary return was displayed in its place. For non-rewarded trials, this was always ‘0 ¢’ and for rewarded trials, this was calculated as the proportion of time spent in the target from the perturbation onset to the trial’s end:

$$return=g∙e^{-\tau p}$$

$$p=1-min\left(\frac{x-x_{0}}{x_{f}-x_{0}},1\right)$$

where x is the time (ms) spent in the target, $x_{0}=500$ is the minimum amount of time (ms) to receive a return, $x_{f}=800$ is the total duration (ms) of the trial, g=15 is the maximum return (¢), and τ is a free parameter adjusted based on pilot data to reduce the discrepancy between easier and harder conditions. In this study, we used τ = 1.428 and τ = 2.600 for an inward and outward perturbation with an outward target, respectively, and τ = 2.766 and τ = 1.351 for an inward and outward perturbation with an inward target, respectively.

The task consisted of 336 trials and was divided into three equal blocks with a free-duration break time between each block. Each block consisted of 112 trials, equally divided between inward and outward perturbation torques, inward and outward target positions, and rewarded and non-rewarded trials. The trial schedule was organized in epochs of 16 trials containing two of each combination of conditions, and the trial order was randomized within each epoch.

For EMG analysis, inward and outward perturbations were used as the contrast to observe the SLR on extensor muscles (Figure 2b). To observe the LLR on the extensor muscles, inward perturbations were used when combined with an outward and inward target (Figure 3a).

#### Proprioception-cued Reaction Time task

The Proprioception-cued Reaction Time task used the same task as the In-Out Target task, with several alterations. First, the background loads were applied to the elbow only, and only outward targets were presented, so that the task consisted of elbow extension movements only. The starting position was located such that the external shoulder angle was 45° relative to the left-right axis, and the external elbow angle was 70° relative to the upper limb. The end target was located at a shoulder and elbow angle of 45°. The perturbation was applied only at the shoulder instead of both shoulder and elbow joints, and the perturbation magnitude was reduced to 0.5 N·m, to ensure that the perturbation led to no significant elbow motion (Pruszynski et al., 2008). Finally, the perturbation time was jittered in a 600–1000 ms window following the target appearance (random uniform distribution). This window was used to measure baseline EMG activity for that trial (referred to as ‘trial baseline’ throughout the text). Participants were informed to initiate a movement to the target as fast as possible following the shoulder perturbation. MTs were defined as the time interval from the perturbation occurrence to entering the end target, regardless of velocity. They performed 27 epochs of 2 rewarded and 2 non-rewarded trials randomly interleaved, resulting in a total of 108 trials per participant. Monetary returns were calculated using the following formula:

$$return=\max\left(g∙scaler∙e^{-\tau p}+shifter,0\right)$$

$$p=\frac{MT}{{MT}_{max}}$$

where MT is the movement time, ${MT}_{max}$ is a normalizing movement time value, g=10 is the maximum amount of money (CA$ cents) that may be earned in a trial, and scaler,shifter,τ are parameters that allow calibrating the return function to typical psychometric performance in the task considered. Their value was determined using pilot data to ensure large variance across participants based on performance and are provided in Table 2.

**Table 2.** Parameters used to compute the return in each rewarded trial, for each condition in the Proprioception-cued Reaction Time, Cursor Jump, Target Jump, and Target Selection tasks.

Task	Condition	Scaler	Shifter	τ	MT_max_ (ms)
Reaction Time	N/A	1	0	2.447	728
Cursor Jump	Inward	0.996	–0.029	4.273	2781
	No jump	0.667	0.079	5.433	2335
	Outward	0.996	–0.041	3.958	2864
Target Jump	Inward	0.999	–0.026	4.281	2697
	No jump	0.683	–0.040	3.893	2882
	Outward	0.999	–0.054	3.853	2690
Target Selection	One target Inward Pert.	0.676	–0.034	6.236	1673
	One target Outward Pert.	0.690	0.004	5.534	2241
	Two targets Inward Pert.	0.749	–0.021	4.904	2373
	Two targets Outward Pert.	0.749	0.009	5.350	2208

For the choice reaction time task, the methods employed are described in Codol et al., 2020a.

#### Target Jump and Cursor Jump tasks

The position of the participants’ right-hand index fingertip was indicated by an 8 mm radius white cursor. At the beginning of each trial, an 8 mm radius start position was displayed at a shoulder and elbow angle of 35° and 65°, respectively, and below it a reward sign showing ‘000’ or ‘$$$’to indicate a non-rewarded or a rewarded trial, respectively. At the same time, a 2 cm radius target appeared at a 55° shoulder angle and same elbow angle as the start position (65°). This yields a reaching movement that consists of a 20° shoulder flexion with the same elbow angle throughout (Figures 6b and 7b6,7). Participants moved the cursor inside the start position, and after 200 ms, +2 N·m shoulder and elbow background torques ramped up linearly in 500 ms. Participants held the cursor inside the start position for 600–700 ms (random uniform distribution), during which baseline EMG activity for that trial was measured (referred to as ‘trial baseline’ throughout the text). Following this, the end target appeared. Participants were instructed to reach as fast as possible to that end target, and that their MT would define their monetary return on ‘$$$’ trials but not ‘000’ trials. They were informed that reaction times were not considered into the calculation of the return. Movement duration was defined as the time interval between exiting the start position and when the cursor was inside the target and its tangential velocity dropped below 10 cm/s. Once these conditions were met, the target turned dark blue, the reward sign was extinguished, and the final monetary return for the trial appeared where the reward sign was located before. In the Target Jump task, during the reach when the cursor crossed past the (invisible) 45° shoulder angle line, the target jumped to ±10° elbow angle from its original position or stayed at its original position (no-jump), with all three possibilities occurring with equal frequency (Figure 7c). In the Cursor Jump task, the cursor position rather than the target jumped to ±10° elbow angle or did not jump.

Monetary return was given using the same equation as for the Proprioception-cued Reaction Time task, with g=10. The parameters used for each condition were calibrated using pilot data to ensure similar average returns and variance across conditions within participants. The values used are provided in Table 2.

Both the target and cursor jumps consisted of 312 trials in one block, equally divided between rewarded and non-rewarded trials, and outward jump, inward jump, and no-jump trials in a 2×3 design. The trial schedule was organized in epochs of 12 trials containing two of each combination of conditions, and the trial order was randomized within each epoch.

For EMG analysis, flexion and extension jumps were contrasted to assess the visuomotor feedback response. No-jump conditions were not used for EMG analysis. All EMG signals were aligned to a photodiode signal recording the appearance of a white 8 mm radius target at the same time as the jump occurrence. The photodiode target was positioned on the screen horizontally 25 cm to the right from the starting position and vertically at the same position as the cursor jumping position (in the Cursor Jump task) or as the target position (Target Jump task). This target was covered by the photodiode sensor and was therefore not visible to participants.

#### Target Selection task

The position of participants’ right index fingertip was indicated by a white 3 mm radius cursor. At the beginning of each trial, a 3 mm radius start position appeared, and a reward sign showing ‘000’ or ‘$$$’ was displayed to indicate a non-rewarded or a rewarded trial, respectively. The start position was located so that the external shoulder angle was 45° relative to the left-right axis, and the external elbow angle was 90° relative to the upper arm. When participants moved the cursor inside the start position the cursor disappeared. It reappeared if the cursor exited the start position before the target(s) appeared. Once inside the start position, the robot applied +2 N·m background torques which were ramped up linearly in 500 ms at the shoulder and elbow to activate the extensor muscles. Then, following a delay of 400–500 ms, a 7 cm radius target appeared at +30° (inward) from the start position (rotated about the elbow joint). In half of trials, a second, identical target also appeared at –30° (outward) from the start position. A jittered 600–1000 ms window followed target appearance, during which baseline EMG activity for that trial was measured. After this, a +2 or –2 N·m perturbation at the shoulder and elbow joints pushed participants away from the start position. Positive and negative perturbations occurred evenly in one- and two-targets trials, yielding a 2×2 task design. For one-target trials, participants were instructed to reach as fast as possible to the target available once the perturbation was applied. For two-target trials, participants were instructed to reach as fast as possible to the target opposite to the perturbation. For example, if the perturbation resulted in an inward push, then the participant should go to the outward target. Therefore, this task design resulted in a co-occurrence of the target selection and divergence of triceps EMG activity compared to one-target trials, enabling us to assess the feedback response that underlies selection of the goal target.

When the (correct) end target was reached, the target(s) turned dark blue, the reward sign was extinguished, and the final monetary return for the trial appeared where the reward sign was located before. For non-rewarded trials, this was always ‘0 ¢’ and for rewarded trials, the return was higher for shorter MTs. MTs were defined as the time interval from the perturbation occurrence to entering the (correct) end target, regardless of velocity. The monetary return equation was identical to that of the reaction time task, with g=15 and the other parameters as provided in Table 2. These parameters were calibrated using pilot data to ensure similar average returns and variance across conditions within participants.

The task consisted of 224 trials and was divided into two blocks with a free-duration break time between each block. Each block consisted of 112 trials, equally divided between one- and two-targets trials, inward and outward perturbation trials, and rewarded and non-rewarded trials. The trial schedule was organized in epochs of 16 trials containing two of each combination of conditions, and the trial order was randomized within each epoch.

### EMG signal processing

For each experiment, the EMG signals of the brachioradialis, triceps lateralis, pectoralis major (clavicular head), posterior deltoid, and biceps brachii (short head) were sampled at 1000 Hz, band-pass filtered between 20 and 250 Hz, and then full-wave rectified. Before each task, participants’ EMG signal was acquired by asking participants to position their arm such that the cursor remained, motionless, at the start position for 2 s (against the background load, if applicable for the task). This was repeated four times, after which the task started normally. Following band-pass filtering and full-wave rectification, the EMG signal of each muscle from 250 ms after entering the start position to 250 ms before the end of the 2 s window was concatenated across all four trials and averaged to obtain a normalization scalar for EMG signals. EMG measures during the task were then normalized by each muscle’s normalization scalar. Follow-up analyses (latency, feedback gains) were performed subsequently on the filtered, full wave rectified and normalized EMG traces.

For all experimental designs using a mechanical perturbation, trial baseline EMG activity was measured in a 50 ms window from 350 to 300 ms before displacement onset, while participants were at rest, background loads were applied, and after targets and reward context were provided. For the Target Jump and Cursor Jump tasks, this was measured in a 50 ms window from 350 to 300 ms before target appearance instead of before displacement onset because movements were self-initiated, and displacements occurred during the movement. However, the same target was displayed in every condition at the start of a given trial in those two experimental paradigms. For all experiments, the trial baseline EMG signals are displayed on a left axis next to the axis showing perturbation-aligned EMG signals. Note that these trial baseline EMG signals are distinct from the four trials described above in this section, which were done before the task started and were used to compute normalization scalars for EMG signals. The trial baseline EMG signals were not used for EMG normalization.

### Statistical analysis

To determine the time at which EMG signals for different task conditions diverged, we used ROC analysis. We used the same approach as in Weiler et al., 2015, using a 25–75% threshold of area under the curve (AUC) for establishing signal discrimination. The threshold was considered reached if two consecutive samples were greater than the threshold value. Discrimination was done for each participant and each reward condition independently, using all trials available for each contrast without averaging. Once the AUC threshold was crossed, we performed a segmented linear regression on the AUC before it crossed the 25–75% threshold. We minimized the sums-of-squared residuals to find the inflexion point, that is, where the two segments of the segmented linear regression form an angle (see Weiler et al., 2015 and the analysis code online for details).

To compute feedback gains, for each feedback response considered, we defined a 50 ms window that started at that response’s latency found for each participant independently using ROC analysis. For the SLR contrast only, we constrained that window to a 25 ms width instead of 50 ms to avoid overlap with the LLR (Pruszynski et al., 2008). We then calculated the integral of average EMG signals in that window using the trapezoid rule (MATLAB’s built-in *trapz* function), for each contrasted condition and each reward value. For instance, for the Target Selection task, the contrasted conditions were trials with an inard perturbation and only one target (no switch), and trials with an inward perturbation and two targets (switch occurring). We then calculated the absolute difference between those two conditions as a measure of feedback gains. We then calculated the log-ratio of the rewarded to non-rewarded conditions as $\log\left(rewardedgain/nonrewardedgain\right)$ . We used ratios to ensure that changes in feedback gains are normalized within participants to EMG activity in the non-rewarded condition. The log function was then applied to linearize the ratio values.

Using a fixed time window to assess feedback gains would allow for a potential confound. If the response onset (latency) is reduced with reward, by definition the EMG signal would be shifted closer to the perturbation onset. Therefore, if the time window used for feedback gains remained in an identical position with respect to the perturbation onset, this is equivalent to assessing feedback gains later with respect to the response onset. From the resulting estimation of feedback gains, one could spuriously conclude that feedback gains are increased with reward, when the gains are merely assessed further away from the response onset, where the EMG signal had more time to diverge.

MTs were defined as the time between the occurrence of the mechanical perturbation being applied and entering the end target in the proprioceptive tasks (In-Out task, Target Selection task, and Proprioception-cued Reaction Time task). They were defined as the time between leaving the starting position and being in the end target with a radial velocity of less than 10 cm/s in the visual tasks.

For the Proprioception-cued Reaction Times task, reaction times were defined as when the (processed) triceps EMG signal rose 3 standard deviations above the trial baseline level (Pruszynski et al., 2008) for 5 ms in a row (Figure 5d). Trials for which the triceps EMG did not meet that criterion were discarded. This represented 91 out of 1836 trials (4.9%). Feedback gains were then estimated using the same technique as for the other tasks, using the reaction time as the starting position for the integral window.

To test for differences between conditions we used Wilcoxon signed-rank tests. For each test, we reported the test statistic W, the effect size r (Kerby, 2014), and the *p* value.

## Data Availability

All behavioural data and analysis code are freely available online on the Open Science Framework website at https://osf.io/7t8yj/. The following dataset was generated: CodolO 2021Sensorimotor feedback loops are selectively sensitive to rewardOpen Science Framework10.17605/OSF.IO/7T8YJPMC991082836637162 The following previously published dataset was used: CodolO 2019Reward-based improvements in motor control are driven by multiple error-reducing mechanismsOpen Science Framework7as8g10.1523/JNEUROSCI.2646-19.2020PMC718975532234779

## Additional files

MDAR checklist
