# Supplementary material for: Sensorimotor feedback loops are selectively sensitive to reward
Source: eLife. 2023 Jan 13;12:e81325. doi: 10.7554/eLife.81325 (PMC9910828; doi:10.7554/eLife.81325)
Supplement: Peer review history

# Editor report: Editor's evaluation

**DOI:** 10.7554/eLife.81325.sa0

**Authors:** Kunlin Wei

**Affiliations:** https://ror.org/02v51f717 Peking University China

The study presents an inventory of behavioral experiments to systematically examine the relationship between motivational reward and motor response to feedback changes. With a unified motor task paradigm, this study offers convincing evidence that the effect of expected reward on sensorimotor feedback depends on the type of feedback response loop involved. These findings can be used as a starting point for exploring neural correlates of reward processing in human motor control.

# Decision letter

**DOI:** 10.7554/eLife.81325.sa1

**Affiliations:** https://ror.org/02v51f717 Peking University China; https://ror.org/02v51f717 Peking University China; https://ror.org/008xxew50 Vrije Universiteit Amsterdam Netherlands

In the interests of transparency, eLife publishes the most substantive revision requests and the accompanying author responses.

**Decision letter after peer review:**

[Editors’ note: the authors submitted for reconsideration following the decision after peer review. What follows is the decision letter after the first round of review.]

Thank you for submitting the paper "Sensorimotor feedback loops are selectively sensitive to reward" for consideration by *eLife*. Your article has been reviewed by 3 peer reviewers, one of whom is a member of our Board of Reviewing Editors, and the evaluation has been overseen by a Senior Editor (Rich Ivry). The following individuals involved in review of your submission have agreed to reveal their identity: Jeroen BJ Smeets (Reviewer #2); Stephen H Scott (Reviewer #3).

Comments to the Authors:

We are sorry to say that, after consultation with the reviewers, we have decided that this work will not be considered further for publication by *eLife*. All reviewers think that systematic evaluation of different feedback processes that are impacted by reward is meaningful and timely for the area of perception and action. However, the reviewers also raised some major concerns that prevent the paper from being considered further.

Specifically, the following two concerns have been raised by reviewers unanimously. 1) The experiments used inconsistent reward function, which affects the acceptance of the paper's general conclusion; 2) The experimental design did not stick to a simple comparison between reward vs. no reward but included confounds other than the availability of reward, especially for the target switch experiment. Given that the study is descriptive without a prior hypothesis and its ambitious goal to comprehensively examine the feedback control in the continuum of feedback latency, we have to caution about the link between the data and the conclusion.

All reviewers' comments and suggestions are attached below. We hope you will find them helpful in furthering your pursuit of the topic.

*Reviewer #1 (Recommendations for the authors):*

How reinforcement impacts motor performance is an active research area that interests many. However, various movement paradigms have been used with various manipulations of reward or punishment. The current study constitutes a timely effort to elucidate possible mechanisms underlying diverse findings in the area. The strength of the paper is that the tasks involving increasingly response latencies are implemented in a single upper-arm experimental setup. The two fastest responses, slow-latency reflex (SLR) and long-latency reflex (LLR), are beautifully examined with a single perturbation scheme. Their related findings are also convincing: SLR was largely unaffected by reward, but the LLR showed a reward-induced increase in EEG gain. The findings that simple reaction time and choice reaction time tasks were improved with reward are replicates of previous research, though the reaction time condition is implemented with a proprioceptive cue here instead of the common visual or auditory cues. However, the other three conditions, i.e., target switch, target jump, and cursor jump, did not yield any behavioral improvements by reward.

My major concern is whether the findings of either presence or absence of reward effect are generalizable and whether uncontrolled confounds can explain them. Note the current paper did not have any prior hypotheses for different conditions; thus, we need to scrutinize the supporting evidence for the conclusions. The study's strength is that diverse upper-arm movement perturbation paradigms are used and systematically varied in response latency, but the weakness also comes with this kind of study design. Each condition used a specific instantiation of each type of feedback control but differed in various factors besides the feedback loops involved. For example, the reward did not improve the performance in the target jump condition but improved the movement time in the target switch condition (though no EMG changes, see below). However, these two conditions had different reward functions, one for minimizing the movement time (MT) and reaction time (RT) but the other for minimizing the deviation from desired movement time. Furthermore, movement directions and muscles examined (brachioradialis vs. pectoralis) differ and probably affect the EMG response that is used for quantifying the reward effect.

Similarly, the cursor jump condition, with a slightly longer latency than the target jump condition but again with a reward function for desired MT, yielded no reward effect either. It makes people wonder whether other task designs would produce the opposite effect on EMG. For example, would the timing and the size of the cursor jump make a difference? What if we reward fast reaction as opposed to maintaining desired movement time in these conditions?

The conditions with significant reward effect are mostly those rewarding faster RT and/or MT; the ones rewarding desired movement time generally returns a null effect. The only exception is the target switching condition, which rewards fast MT and shows no reward effect. However, the target switch perturbation is associated with a peculiar instruction: once perturbed, the participants were required to relax their arms and let the perturbation push them toward the target and stop there. Relaxing while stopping at a target might conflict with the rewarding goal to move fast. Besides the instruction differences, the conditions drastically differ in muscles examined, movement amplitude/timing and etc. These differences make the conclusions, based on a single specific instantiation of each feedback control paradigm (using the taxonomy by Stephen 2006), debatable.

Relatedly, the lack of reward effect in the target switch, cursor jump, and target jump conditions is taken as evidence that feedback responses that rely on sensorimotor and premotor cortices are not modulated by reward, but those relying on prefrontal cortices are. However, it is not clear to me why the LLR condition involves prefrontal associative cortices, but the target jump condition does not. I did not find a discussion of this selective involvement of brain regions either. Given the concern that the specific task designs might cause those null effects, it might be premature to draw this conclusion.

The second major concern is whether analyzing a single muscle adequately captures the perturbation effect and the reward effect. For example, the reward improved the performance in the target jump condition (figure 3g), but there is no EMG difference. This has been attributed to other feedback responses that may not be apparent with the task contrast here. But looking at Figure 3J, there is no EMG activity difference between the reward and the control conditions whatsoever. Then, how can the immediate result of EMG, i.e., the movement time, differ between conditions? Is it possible that the muscle activity examined is not relevant enough for the task? This relates to a methodological issue: is the null effect of EMG response to reward caused by the selection of muscles for analysis? For example, the target and cursor jump conditions select the pectoralis muscle only, and thus only leftward target jumps and right cursor jumps are used for analysis. This is reasonable as the pectoralis muscle directly relates to these perturbation directions, but these perturbations probably cause changes in other muscles that are not examined. How can we be assured that any reward effect that is really there is all captured by analyzing the pectoralis only?

For the questions raised above, I would suggest:

1) Design tasks with similar reward functions, at least.

2) Analyze more muscles.

3) Explain why some tasks rely on associative cortices while others on premotor and sensorimotor cortices.

4) Solve the issue of the conflicting instructions for the target switch condition.

*Reviewer #2 (Recommendations for the authors):*

It is known that one can obtain a reward, motor performance improves. The authors' aim is to answer the question "which of the nested sensorimotor feedback loops that underly motor performance is/are affected by expected reward.?"

The authors provide a large set of experimental conditions and show that their manipulation of the reward affects the response to some of the perturbations.

A major weakness is that the paper lacks a clear hypothesis on how reward would affect the feedback loops. There are several possibilities. It could speed up the information processing, increase the gain, etc. Without a clear hypothesis, it is unclear what the differences are one should be looking for. The authors instead perform a fishing expedition and look for any difference.

A second major weakness is that the conditions differ not only in the aspect that is presented as the reason for performing the task but also in several additional aspects. For instance, the paper contains two reaction time tasks. One is based on visual input, the other on proprioceptive input. However, the visual one is also a choice reaction time, whereas the proprioceptive one is not. The most serious variation is that what the authors reward differs between the experiments. For instance, performance in the target-switch condition is rewarded for short movement times whereas small deviations from the desired movement time are rewarded in the target-jump condition. In other conditions, the reward is based on time-in-target. So, any difference between the experiments might be due to this difference in reward criterion.

A third major weakness is that the authors use 'feedback' for aspects of control that are feedforward. Feedback control refers to the use of sensory information to guide the effector to the target. However, switching to another target (second experiment) is a decision process (selecting the goal), and is not related to reaching the target. It is unclear how this relates to the "target jump" condition, which can be interpreted as resulting from feedback control.

A fourth major weakness is that the analysis (or the written report of it) is sometimes confusing. For instance, the authors use terminology R1, R2, R3 as defined by Pruszynski et al. (2008). They don't report the definitions themselves (e.g.: R2 corresponds to 45-75 ms after the perturbation). Despite explicitly citing this paper, they don't seem to use these definitions. Instead, they redefine them as "R2 and R3 epochs were taken as the first 25 ms.… after each participant's LLR latency". By using this flexible definition of epochs, the epoch is not an independent variable anymore.

A fifth major weakness is that it is unclear in the SL/LL experiment whether the stimulus (the stretch of the muscle) is affected by the reward, as the mechanical stiffness of the muscle might have been affected by the expected reward (e.g. by co-contraction).

There are at the moment some conflicting views on the relationship between reward and motor variability and learning. Well-designed experiments would be able to help to advance the field here. As the authors varied much more between the experiments than only the loop involved, they have not convinced me that the differences they report are indeed related to differences in how expected reward affects the nested sensorimotor feedback loops.

Using page numbers would have facilitated feedback

Title: In my understanding, it should contain "expected" rewards, as the responses that are investigated occur before the reward is provided.

"a 10 cm target appeared at 20 degrees" Use comparable units (all degrees or all cm) and clarify whether the size is radius or diameter.

A figure explaining the time-course of a trial might be helpful.

"an inward" Better use "(counter-)clockwise"

In several captions it is mentioned: "The left panels show EMG at trial baseline (see methods)", but in the methods, there is no mention of "trial baseline". There is mention of a "mean baseline scalar" and that "EMG measures during the task were then normalised by each muscle's baseline scalar." I have no idea what this means. Is the scalar subtracted, expressed in multiples of baseline activity? And are the plotted signals those after normalising?

It is unclear how latencies and reaction times are determined. There are many options for this, and the results of the analyses of latencies depend critically on which options are chosen.

"Conversely, the LLR arose.… (LLR not occurring, Figure 2c)." This is not my interpretation. In both cases, an LLR is present, in one case much stronger than in the other. Secondly, the effect of the task is not present at the onset of the LLR, but starts at a moment the LLR has already started. The authors refer to this latter time as the latency, but the figure shows a clear SL and the onset of the LL, which is clearly before the effect kicks in.

Figure 2: explain graphically what continuous and dashed lines signify, and green/purple. I can't follow panel d: In my understanding, SLR and LLR are determined by subtracting data from within the same experiment in a different way. How can this have (for at least one participant) such a large effect on the difference of time-on-target between rewarded and non-rewarded trials? How do the data in panel f link to those in panel e?

"For this analysis, we did not use a log-ratio" This is not clear to me. You normalised the EMG and expect a change in gain. So why not a log(ratio)?

It would help if all the figures were showing similar data in the same format. The various ways to plot data are confusing.

Please add plots of the displacement as a function of time. Specify in caption whether the EMG plots show the means of all participants or of a typical single participant

Please make sure that all major aspects of the task are mentioned in the results text. Now the most essential information (what is rewarded in each experiment) is missing, whereas total irrelevant details (that no-reward corresponded to 0 ¢ CAD) are provided. Additionally, understanding why mechanical perturbations are provided as torques (and not as displacements) might be easier to follow if you briefly mention in the Results section that an exoskeleton is used.

Figure 1a is very useful to help the reader to understand the authors' line of thought. Unfortunately, the authors don't lead the reader through this figure. As latencies relate to the hierarchy, it might be simpler to add the various loops from panel b to panel a, and remove panel b.

"Codol et al. (2020)." Is it 2020a or 2020b?

I am not sure where (Dimitriou et al. 2013) claimed that responses to a cursor jump have a longer latency than to a target jump (section "Online visual control of limb position was also unaltered by reward"). In an earlier study, the opposite has reported (Brenner and Smeets 2003), which is in line with the scheme in figure 1a.

What are 'goal-sensitive' feedback responses? What is 'absolute response latency'? These concepts are not explained.

Please be consistent. The authors use, 'stretch reflex" and "short-latency reflex" interchangeably. In the abstract and discussion, the authors refer to "eight different kinds of sensorimotor feedback responses". In figures 1a and 6a, I count nine kinds of responses. What happened to the ninth one? In table 1, I count 5 tasks. Please provide a mapping from tasks to responses. Secondly, provide for all experiments similar plots. Now the exoskeleton that is very relevant is not drawn, but the endpoint-Kinarm that is not essential is drawn.

Discussion: this section contains an aspect that could have been discussed in the introduction (cortico-cerebellar loops not assessed), as this is not related to the results or conclusions. I miss a discussion of how behaviour can be improved by expected reward with such little changes in the underlying sensorimotor control. A last item that could be discussed is that reward might affect behaviour not only by expected reward but also through a learning mechanism, so the (lack of) reward will affect the trial after the (lack of) reward.

References

Brenner E, Smeets JBJ (2003) Fast corrections of movements with a computer mouse. Spatial Vision 16:365-376 doi: 10.1163/156856803322467581

Dimitriou M, Wolpert DM, Franklin DW (2013) The Temporal Evolution of Feedback Gains Rapidly Update to Task Demands. Journal of Neuroscience 33:10898-10909 doi: 10.1523/jneurosci.5669-12.2013

Pruszynski JA, Kurtzer I, Scott SH (2008) Rapid motor responses are appropriately tuned to the metrics of a visuospatial task. Journal of Neurophysiology 100:224-238

*Reviewer #3 (Recommendations for the authors):*

The question on how reward or value impacts feedback processes is important to understand. Previous studies highlight how reward impacts motor function. Given feedback is an important aspect of motor control, it is useful to know which feedback responses may be specifically impacted or altered by reward.

A clear strength of the manuscript is the systematic evaluation of different feedback processes reflecting different latencies and behavioural contexts to initiate a response. These differences reflect differences in the underlying neural circuitry involved in each of these feedback processes. Examination on how reward impacts each of these processes using similar techniques and approach provides a comprehensive examination on how reward impact feedback responses and a much cleaner overview of the problem, rather than a fractured examination if explored over many separate studies.

The manuscript uses a functional taxonomy suggested by Scott (2016) to define the behavioural contexts examined in the paper. In most cases, the experimental paradigms match these taxonomies. However, some confusion seems to occur for responses elicited at ~50ms following mechanical disturbances which includes two distinct types: 1) goal-directed online control and 2) triggered reactions. These two conditions are behaviourally quite different as the former maintains the same goal before and after the disturbance, whereas the latter switches the behavioural goal, and thus, feedback responses are now set to a new spatial goal. Triggered reactions are examined in the present study, but it is assumed that this reflects goal-directed online control (the former). Thus, responses at ~50ms can reflect substantially different behavioural conditions (and likely processes) and these distinctions should be recognized.

I think the simplest approach for quantifying the impact of reward on corrective responses is to compare corrective responses in a single condition with and without reward. However, the manuscript used paired behavoural conditions to look at the difference in EMG between contexts and then identify if this EMG difference changes between rewarded and non-rewarded trials. This makes the material more complex to understand and follow. Admittedly, the use of this EMG difference between conditions works well if reward should increase a response for one context and decrease it in the other. For example, target jumps to the left compared to the right increase pectoralis activity for the leftward jump and decrease for the right jump. Reward should enhance both of these reciprocal responses (increase the first and/or decrease the latter) and thus lead to a larger EMG difference for rewarded trials. So this contrast approach makes sense in that experiment. However, the contrast for goal-tracking (actually should be called goal-switching) experiment contrasts the switching goal condition with a control condition in which corrective responses were generated to the original spatial goal. In this situation, both contexts could show an increase in EMG with reward, and in fact, that appears to be the case shown in Figure 3e (top panel shows both conditions have a slight increase in EMG for rewarded trials). However, by looking at the difference in EMG between conditions, this reward-related activity is removed. I think these two behavioural contexts should be assessed separately. Critically, the baseline condition where corrective responses were generated to the original goal fills the void regarding goal-directed online control mentioned in the previous paragraph that occurs at ~50ms. If there is a significant change in EMG for the goal-directed online control, then it could be used as a contrast for the target switching task to address whether there is any greater increase in EMG response specifically related to target switching.

I think there is some confusion with regards to some of the functional taxonomy and the experimental paradigms to assess these processes from the functional taxonomy outlined in Scott (2016). Specifically, there are two distinct behavioural processes that occur at ~50ms related to proprioceptive disturbances: there is 1) online control to an ongoing motor action where the behavioural goal remains constant, and 2) triggered reactions to attain a new goal. The present study is the latter and was developed by Pruszynski et al., 2008 (should be cited when describing the experiment) and is really just a spatial version of the classic resist/don't resist paradigm. However, this Target In/Out task is assumed to be the former both in Figure 1 and the text. These are distinct processes as the goal remains constant in the former and switches in the latter. The former is comparable to a cursor jump task where the arm (or cursor) is shifted, but the goal remains the same. I think Figure 1 and the text needs to recognize this distinction as outlined in Scott (2016).

This distinction between triggered reactions and online control is important as triggered reactions are related to another task examined in this study, proprioception-cued reaction time task. These are essentially the same tasks (disturbance drives onset of next action in Scott 2016), as the only difference between them is the size of the disturbance with triggered reactions using large disturbances leading to responses at ~50ms and small disturbances for reaction time tasks leading to responses starting at ~110ms. These are likely not distinct phenomena, but a continuum with the latency and magnitude likely impacted by the size of the mechanical disturbance, although I don't think it has ever been systematically examined. Critically, they are very similar from a behavioural context perspective. Interestingly, the present study found that reward shortened the latency and increased the magnitude for the proprioceptively cued reaction time task and increased the gain for the triggered reaction, but not the latency likely due to the fact the latter hit transmission limits. The manuscript should recognize the commonality in these behavioural tasks when introducing the tasks. Perhaps these experiments should be grouped together. I think the strategy of the manuscript was to introduce experiments based on their latency, but this creates a bit of an artificial separation for these two tasks.

It would be useful to add a goal-directed online control experiment to assess EMG responses when reaching to spatial goals with mechanical disturbances with and without reward. This would provide a nice parallel to the experiments examining cursor jumps to explore online control of the limb. Previous work has shown that increases in urgency to respond to a postural perturbation task led to increases in long-latency responses (Crevecouer et al., JNP 2013). Urgency in that study and reward in the present study are related as reward was based on how long individuals remained at the end target which is similar to the need to return faster to the target in Crevecouer et al. There may be specific differences between posture and reaching, but the basic principle of corrective responses to attain or maintain a goal are similar. In fact, you second experiment incorporates a simple goal-directed online control task with mechanical disturbances in the goal-tracking task displayed in 3a. This could be analyzed on its own to fill this void.

The experimental paradigms use comparisons between two conditions (termed a control and a manipulation condition, in some cases). I'm not entirely sure why they did this as a simpler strategy would be to examine the differences between rewarded and unrewarded trials for a given condition. The logic (and value) may be that multiple feedback processes could be impacted by reward and you wanted to see the incremental change between feedback processes. However, looking at the difference in EMG in the goal-tracking task makes me wonder if the authors missed something. It looks like both the control and manipulation condition show a slight increase in EMG in Figure 3e. However, the statistical test simply looks at the difference in these responses between control and manipulation, and since both show a slight increase for rewarded trials, the difference in EMG removes that increase observed in both signals resulting in no difference between rewarded and non-rewarded trials. I think the control and manipulation conditions should be separated as I don't think they are directly comparable. While lines overlap in the top panel of Figure 3e, it looks like the rewarded trials for the target switch condition may show a reduction in time and an increase in magnitude during the LLR for rewarded trials (the challenges of a dashed line).

The online control condition from the target switching task (Figure 3a) could be examined on its own. If a contrast design was important, that would require pairing the resistive load with an assistive load, or perhaps loads to deviate hand orthogonal to the target direction to parallel the cursor jump experiment.

I think it's confusing to frame the first two experiments based on short-latency, long-latency, and slower long-latency. You have provided a clean approach to introduce different feedback processes based on behavioural features of a task (Figure 1). I think you should stick to these behavioural features when describing the experiments and not talk about short-latency, long-latency and slower long-latency when developing the problem and experiments as this confuses the taxonomy based on behaviour with time epochs. As you carefully point out, there are many different layers of feedback processing and so epochs of time may include influences from many pathways and two processes may happen to take the same time (i.e. goal-directed online control and triggered reactions). Further, there are two distinct processes at ~50ms which needs to be clarified and kept distinct. Thus, behavioural context and not time epoch is important to maintain. This is why the later experiments on cursor jump, target jump and choice reaction time are much easier to follow.

The impact on reward on baseline motor performance is a bit confusing. It is not clear that various statistics of motor performance in the various conditions is related to the baseline movements without disturbances or with disturbances. This may be stated in the methods but should be clearly stated in the main text and legends to avoid confusion.

I don't think it is useful to talk about goal-tracking responses for experiment 2 as the term tracking is usually reserved for moving targets. I kept re-reading trying to understand how the goal was moving in the task and how the individual was tracking it, but this clearly didn't occur in this experiment! Rather, this task is probably best characterized as goal switching (as stated in methods section). The term slower in the title is also unnecessary and confusing. Again, stick to behavioural features of the task, not time epochs.

[Editors’ note: further revisions were suggested prior to acceptance, as described below.]

Thank you for resubmitting your work entitled "Sensorimotor feedback loops are selectively sensitive to reward" for further consideration by *eLife*. Your revised article has been evaluated by Timothy Behrens (Senior Editor) and a Reviewing Editor.

The manuscript has been improved but there are some remaining issues that need to be addressed, as outlined below:

All reviewers find this paper (or a revised version of the previous submission) a timely effort to study how the motivational factor affects motor control. The methodological improvements are also helpful in addressing previous concerns about consistency across experimental tasks. However, the reviewers converged on two major concerns:

1) The link between functional tasks and their assumed feedback loops is poorly specified. For example, goal-switching and online action selection appear the same in function. Target jump and cursor jump are also similar. The LLR contrast and the target selection yielded similar latency and had the same sensory input but were treated as involving different feedback loops. The functional taxonomy proposed in Scott 216 paper was not meant to suggest that each functional process was supported by a different "feedback loop." Thus, we suggest that the introduction should be less conclusive about the functions that the various experimental paradigms address and whether they tap into different or the same feedback loops. Perhaps simply referring to the existence of these paradigms in the introduction is enough for this descriptive research. At the same time, the link between functional tasks and their assumed feedback loops should be discussed more appropriately in the discussion.

2）The critical methodological problems that might affect the validity of the findings should be addressed. These problems include how the latency is determined (e.g., the need to have other methods to confirm the latency estimation, the need to have a fixed time window), which segment of EMG should be analyzed (e.g., R2 and R3 splitting), and which muscles should be used for analysis (e.g., most analyses based on one muscle though all muscles are monitored; having an arbitrary choice of muscles is a warning sign for p-hacking). These problems are more detailed in Reviewer 3's comments.

*Reviewer #1 (Recommendations for the authors):*

The study aims to test whether sensorimotor feedback control is sensitive to motivational factors by testing a series of tasks that presumably rely on different feedback loops. The first strength of the study is that all the feedback control tasks were designed with a unified upper-limb planar movement setting with various confounding factors under control. Its previous submission a year ago had received some major criticisms, mostly about inconsistency across tasks in task goals, analyzed muscles, and reward functions. The new submission has used re-designed experiments to keep consistency across tasks and successfully addressed most, if not all, previous major concerns. As a result, this study gives a more complete picture of how motivation affects feedback control than previous studies that did not scrutinize the feedback loop involved in the task.

The study found that the fastest feedback loops, both for visual and proprioceptive feedback, are free from the effect of reward in terms of muscle response. The earliest reward-sensitive feedback loop has a latency of about 50ms, depicted by the response to the proprioceptive perturbation. Reduced response latency and increased feedback gains underlie the reward-elicited improvements, but their roles vary across tasks.

The weakness of the study is that the underlying mechanisms for the heterogenous results are speculative. Though the study included five tasks and one previous dataset, it did not conduct experiments for some tasks, or failed to have electromyography measurements. These tasks include those related to vision-cued reaction time, alternative targets, and choice RT. The incomplete task set might prevent drawing conclusive explanations for the current findings. The theoretical account to explain the increased feedback gain is so-called anticipatory pre-modulation, but this term is unspecified in any detail based on the present findings. Using this account to explain the finding that the cursor jump task (in contrast to the target jump) failed to induce a reward effect in feedback gain, the authors hypothesize that the anticipatory pre-modulation does not work for the cursor jump task since it cannot prime the participants with the probability of a cursor jump. I find this explanation unsatisfactory: the probability of the jump to either direction is similar for both the target jump and cursor jump tasks as they share identical trial designs.

In sum, the study achieved its goal of testing whether the motivation factor improves feedback control when different feedback loops are predominantly involved in various tasks. The experimental tasks are carefully designed to avoid multiple confounding factors, the analysis is solid, and most of the results are convincing (with the exception of the "significant" difference in Figure 5f). The study aim is more explorative than hypothesis-driven, thus limiting the insights we can obtain from the heterogeneous results. However, through systematically studying feedback loops in a unified experimental framework, the study provides more insights into the effect of motivation on sensorimotor feedback control in the aspect of response latency and gain and thus can serve as a new stepping stone for further investigations.

Labeling the experiments with numbers would be good, especially considering the paper also includes an online dataset (Figure 5g and 5h).

Page 5: "Next, we assessed the time of divergence of each participant's EMG activity between the reward and no-reward conditions using a Receiver Operating Characteristic (ROC) signal discrimination method…"

Does this refer to the divergence of EMG activity from the baseline, not between the two conditions?

Figure 2b: what do the two colors mean? Reward and no reward?

Figure 5f: though it is statistically significant, 8 out of 17 subjects showed increased (or unchanged) RT as opposed to reduced RT.

Figure 6: Is the MT improvement a result of a movement speed increase not related to the cursor jump response that happens during the movement?

The target jump condition is theorized with longer latency than the cursor jump condition (Figure 8). Is this really the case? It appears that their RTs are similar.

The paper proposes to classify feedback control by sensory domain and response latency, not by function. The argument is that "…it does not macth any observed pattern here (Figure8)". But what pattern does this refer to? The fact that response latency and modality matter for the "reward effect" does not justify ditching the use of "function." In my opinion, the more apparent pitfall would be the loose use of "function" terms for different tasks. For instance, I wonder whether associating the target jump task with online tracking of the goal is reasonable. Tracking is typically referred to as using an effector to follow or spatially match a moving visual target. It is certainly not the case for a reaching movement to a possibly-changing target that has not been attained yet. It appears to me that for the same function, people can design drastically different tasks; that's the real problem that the current study should emphasize.

*Reviewer #2 (Recommendations for the authors):*

The question on how reward or value impacts feedback processes is important to understand. Previous studies highlight how reward impacts motor function. Given feedback is an important aspect of motor control, it is useful to know which feedback responses may be specifically impacted or altered by reward.

The manuscript uses a functional taxonomy suggested by Scott (2016) to define the behavioural contexts examined in the paper. A clear strength of the manuscript is the systematic evaluation of these feedback processes with distinct latencies. This resubmission addresses several issues raised in the initial review. Notably, most experiments have been redone to better align with the defined behavioural processes and the use of more standardized experimental approach and analyses techniques across experiments.

There are some methodological issues that are either poorly described or seem to be a problem. From the methods section, it looks like only the R2 and R3 epochs (50 to 100ms) were examined for each experiment. This doesn't make sense for experiments such as target and cursor jumps that only lead to EMG responses at ~100ms after the disturbance. As well, magnitude changes are monitored for 4 different muscles, but why is there only one latency examined (last panels in most figures) and which muscle is being used is not clear for each experiment.

I think some of the points raised in the discussion need to be developed more including the addition of pertinent literature. Specifically, the section on 'categorizing feedback control loops', brings up the point that it might be better to not use functional processes as a framework for exploring feedback control. Instead, they suggest categorization should be based on neural pathways, neural regions and sensory modalities. There are no citations in this section. However, in the conclusion it appears they suggest this paragraph is about using a bottom-up approach based on pathways and neural regions rather than a top-down functional approach. If that is their message, then the bottom-up approach has been around since Sherrington (see also recent ideas by G. Loeb) and so it would be worthwhile to include some integration of existing ideas from the literature (if they are related). While this is a worthwhile conversation, I think the authors should be cautious in concluding from this one behavioural study on reward that we should just ignore functional processes. Perhaps the problem is the term of linking complex functional processes to single 'feedback loops' as such processes likely engage many neural pathways Notably, the present discussion states that the cortico-cerebellar feedback loop was not considered in the present study. However, it likely was involved. In fact, in the 1970s the R3 response was commonly associated with the cerebellar-cortical feedback pathway. The richness of brain circuits engaged after 100ms is likely substantive. Thus, there needs to be some caution on linking these behavoural experiments to underlying brain circuits. The value of thinking about behavioural function is not because function can be found in a single brain region or pathway. Rather it is to ensure tasks/experiments are well defined, providing a sound basis to look at the underlying circuits and neural regions involved.

From above, the key points I think need to be considered are defining the time epochs under study for each experiment (need to ensure reader knows for each experiment) and why latency in only one muscle and which one for each study. The other point is to expand section on categorizing feedback loops with the existing literature, as suggested above.

The diagrams are very well organized. However, I wonder if it would be useful to show the hand speed against time to highlight your point that movement times were faster in rewarded trials in either Figure 1 or 2. This may not be necessary for all figures, but the first few to give the reader some sense of how much hand speed/movement time was altered.

*Reviewer #3 (Recommendations for the authors):*

It is known that if one can obtain a reward, motor performance improves. The authors' aim is to answer the question which of the nested sensorimotor feedback loops that underly motor performance is/are affected by expected reward (and how).

The authors provide a large set of experimental conditions and show that their manipulation of the reward affects some aspects of the response to the perturbations in a latency-dependent way. The experiments are designed very similarly, so easy to compare. The authors succeed to a large extent in showing very convincingly that reward affects some feedback loops, but not others. However, there are some weaknesses, mainly in how the authors deal with the possibility that latencies might depend on reward. If this is the case, then the analysis becomes problematic, as the way the gain ratio is defined (based on differences) assumes equal latencies. The authors do not have a solid method to disentangle effects on latency from effects on gain.

A weakness is that there is no clear theory to identify feedback loops. The most evident example is the use of the functions (the colour code in Figure 1). For instance, what is the difference between 'goal-switching' and 'on-line action selection'? To me, these two refer to the same function. Indeed, the latencies for on-line goal switching depend on the design of the experiment, and even be as short as those for on-line tracking of the goal (Brenner and Smeets 2022). Also, the difference in labeling the SLR and LLR is not straightforward. In figure 2, it is clear that there is a LL reflex that depends on reward, the function here is on-line control of the limb. In the experiment of figure 3, that also yields a LLR, I see no reason why the function would not be the same, despite the task being different. The splitting of the LLR in a R2 and R3 makes things even more complicated. Lastly, it is interesting that the authors label the feedback loops involved in experiment 3 to differ from those in experiment 2, although they have the same latency and same sensory input.

A second weakness is the discussion on the latency of the responses. We have shown previously that conclusions about effects of a manipulation on latency depend critically on the way latency is determined (Brenner and Smeets 2019). So the effect of reward on latency might be an artifact, and should be confirmed by using other methods to determine latency. The authors argue in their rebuttal against using fixed time-windows. I am not convinced for 3 reasons: 1) by using a data-driven definition of the various reflex-epochs, the authors compare responses at different moments after the perturbation. We see for instance in figure 2h that the latency obtained for a single participant can differ 20 ms between the rewarded and non-rewarded condition (without any meaning, as the two conditions have the same latency, and the length of the arm was also not changed), so that the gain compares different epochs without any reason. Thus any uncertainty in the determined latency affects the values obtained for the gain-ratio. 2) the paper that defined these epochs (Pruszynski et al. 2008) used fixed periods for R1, R2 and R3. 3) the much older monkey-work by Tatton et al. reported consistent latencies for R1 and R2, and only variable latencies for R3. The authors do the opposite: assume a fixed latency of R3 (relative to R20, and variable for R1 and R2.

A third weakness is that the authors seem to claim that the changes in the feedback are causing better performance. The first aspect that troubles me is that only one factor of performance is provided (speed), but higher speed generally comes at the cost of reduced precision, which is not reported. By the way, MT (i.e. end of movement) is not defined in the paper. The second aspect is that I think they are not able to determine causality using the present design. The authors do not even try to show that feedback and MT are correlated. The authors should then limit their claim to their finding that reward changes movement time and feedback mechanisms.

A fourth weakness is their flexibility in the choice of their dependent measures, and (related) the excessive use of hypothesis testing (p-values). For instance, they measure the EMG from five muscles, and use sometimes all signals, and sometimes restrict themselves to the ones that seem most suited (e.g. when claiming that the latency is significantly reduced). Not analysing some signals because they are noisier gives an impression of p-hacking to me. Furthermore, by using more than one signal to test a hypothesis about a feedback loop, they should use a (Bonferroni?) correction for multiple testing. By reporting p-values rather than the differences themselves, the authors obscure the sign of the difference. A better strategy would be to report all differences with their confidence intervals and base your conclusion on this (the reader can check to what extent this ensemble of results indeed supports your conclusion).

References

Brenner E, Smeets JBJ (2019) How Can You Best Measure Reaction Times? Journal of Motor Behavior 51:486-495 doi: 10.1080/00222895.2018.1518311

Brenner E, Smeets JBJ (2022) Having several options does not increase the time it takes to make a movement to an adequate end point. Experimental Brain Research 240:1849-1871 doi: 10.1007/s00221-022-06376-w

Pruszynski JA, Kurtzer I, Scott SH (2008) Rapid motor responses are appropriately tuned to the metrics of a visuospatial task. Journal of Neurophysiology 100:224-238 doi:10.1152/jn.90262.2008

The authors might want to add information on the correlation between changes in feedback gain/latency and changes in MT.

P2 "More recent studies outline" The studies that follow aere not more recent tghan the ones in the previous paragraph

Figure 2b: explain somewhere how the trajectories are averaged. As the response latencies might vary from trial-to-trial, averaging might introduce artifacts. Explain the method, and indicate in the bottom half of the plot which of the 15 curves belongs to the participant shown in the upper half.

Figures 2d,e, 3d,e, etc: Unclear why the left panels with the trial-baseline are included, as it is visible in the right panels as well (from -50 to 0). In the right panel, use the same x-axis, so responses are more easily comparable. Please indicate the time-window under study by a bar on the time-axis. I understand that the time-window used varies a bit from participant to participant, you might show this by letting for instance the thickness or saturation of a bar at each time indicate the number of participants that contributes to that part. Also: use milliseconds to report the difference in MT.

Figure 2f: The caption text "Feedback gains following SLR onset" is not informative and even wrong. It is a ratio, and it is from a limited time-widow.

Statistical reports in the text makes reading hard (e.g. on page 5 21 times an "="). Try to move these numbers to the figures or a table.

Make sure that you use similar phrases for similar messages. E.g., the analysis of MT in 2.1 is described totally different from that in 2.2, whereas the analysis is the same. In a similar fashion, don't use "Baseline EMG" for two different measures (the one based on 4 separate trials, and the one based on al trials).

P7: The authors report separate values for the gain-ration for the R2 and R3 epochs, but don't show these, bit only a single combined ratio.

P8, figure 3d (lower): how is it possible that we see that the green curve responds clearly earlier than the purple, but we do not see this in figure 3i?

P9 (figure 4): I am puzzled by the relation between panel e and f. Panel e looks very similar to the corresponding panel in figure 3 (green clearly different from purple), but the sign of the gain log-ratio is opposite to that in figure 3.

It is confusing to redefine the concepts 'R1', 'R2", and 'R3'; in the present paper these refer to variable intervals that depend on the participant, whereas the paper that defined these intervals (Pruszynski et al. 2008) used fixed intervals.

P26 "we defined a 50 ms window" What is defined as 50ms? The R1 is only 25, and the complete set R1-R3 is 75 ms.

P28 De reference to De Comité et al. 2021 is incomplete. I guess you want to cite the 2022 eneuro paper.

P32 De reference to Therrien et al. 2018 is incomplete. I guess you want to cite their eneuro paper.

# Reply: Author response

**DOI:** 10.7554/eLife.81325.sa2

[Editors’ note: the authors resubmitted a revised version of the paper for consideration. What follows is the authors’ response to the first round of review.]

Comments to the Authors:

We are sorry to say that, after consultation with the reviewers, we have decided that this work will not be considered further for publication by eLife. All reviewers think that systematic evaluation of different feedback processes that are impacted by reward is meaningful and timely for the area of perception and action. However, the reviewers also raised some major concerns that prevent the paper from being considered further.

Specifically, the following two concerns have been raised by reviewers unanimously. 1) The experiments used inconsistent reward function, which affects the acceptance of the paper's general conclusion;

We re-designed and re-collected 4 experimental designs out of 5. The experiment that we kept was the first experiment described in the study, which quantified the short- and long-latency stretch reflexes, and is now referred to as the “In-Out Target” task to match previous literature. The four new experiments were all designed to have a similar reward function to the experiment that was kept identical to the first version of this study. We decided in favour of keeping the reward function of the first experiment because that experiment consistently appeared from reviewers’ feedback as the most compelling, and the reward function of the other experiments raised potential confounds as pointed below in the second main concern given by reviewers. On the other hand, the reward function of the first experiment did not give rise to these concerns about confounding factor (see below).

2) The experimental design did not stick to a simple comparison between reward vs. no reward but included confounds other than the availability of reward, especially for the target switch experiment. Given that the study is descriptive without a prior hypothesis and its ambitious goal to comprehensively examine the feedback control in the continuum of feedback latency, we have to caution about the link between the data and the conclusion.

We listed the potential confounds raised in the individual reviews:

- Potential Confound 1: The main muscle used for analysis differed from experiment to experiment.
- Potential Confound 2: The reward function was different from experiment to experiment.
- Potential Confound 3: The target switch task required participants to “let go” along the perturbation during the main condition, while all other experiments conversely required the participants to “move as fast as possible” against the perturbation and to the target.
- Potential Confound 4: The target switch task was the only task where the proprioceptive cue was provided during movement rather than during postural control.

Overall, through re-designing and re-collecting 4 of the 5 experiments, we harmonized the specific muscles on which the analyses are based, now use the same experimental apparatus for all experiments, and we keep consistent the reward/reinforcement schedule across all experiments. We also include analyses of more muscles involved in the experimental tasks.

To address individual review comments (comments which were not shared across all reviewers), we edited the graphical summary figures to provide a more consistent overview of existing literature, and we enhanced and expanded all figures showing empirical results to improve completeness and readability of the data and task designs. We also completely re-wrote the discussion to address remaining questions, re-focus the points already discussed and improve the logical structure. Finally, we included missing information in the methods, figure captions, and references, and removed ambiguous or misleading wordings that were pointed out.

Finally, based on the points raised by the reviewers below and changes in the task designs, we adjusted the terminology of some elements within the manuscript.

- The “target switch” task is now renamed “target selection” task.
- “Stretch reflex” is now renamed “rapid response” to match the terminology used in one of the original studies that we are building from (Pruszynski et al., (2008)).
- The task emphasizing the SLR and LLR responses is now labelled “In-Out Target” task to match the terminology of the original study that designed and used this task (Pruszynski et al., (2008)).

Beyond the above shared concerns, individual concerns were raised by each reviewer, which we answer below.

Reviewer #1 (Recommendations for the authors):

How reinforcement impacts motor performance is an active research area that interests many. However, various movement paradigms have been used with various manipulations of reward or punishment. The current study constitutes a timely effort to elucidate possible mechanisms underlying diverse findings in the area. The strength of the paper is that the tasks involving increasingly response latencies are implemented in a single upper-arm experimental setup. The two fastest responses, slow-latency reflex (SLR) and long-latency reflex (LLR), are beautifully examined with a single perturbation scheme. Their related findings are also convincing: SLR was largely unaffected by reward, but the LLR showed a reward-induced increase in EEG gain.

Considering this statement (amongst others), we decided to design the new experiments to be closer to this experimental design, particularly the target switch experiment.

findings that simple reaction time and choice reaction time tasks were improved with reward are replicates of previous research, though the reaction time condition is implemented with a proprioceptive cue here instead of the common visual or auditory cues.

The results of the new experiment testing for proprioception-cued reaction times now replicate this result. The main change in the new design is that the movement participants are required to initiate emphasizes lateral triceps contraction (a forearm extension) instead of brachioradialis contraction (a forearm flexion). This is done to address Potential Confound 1.

However, the other three conditions, i.e., target switch, target jump, and cursor jump, did not yield any behavioral improvements by reward.

My major concern is whether the findings of either presence or absence of reward effect are generalizable and whether uncontrolled confounds can explain them. Note the current paper did not have any prior hypotheses for different conditions; thus, we need to scrutinize the supporting evidence for the conclusions. The study's strength is that diverse upper-arm movement perturbation paradigms are used and systematically varied in response latency, but the weakness also comes with this kind of study design. Each condition used a specific instantiation of each type of feedback control but differed in various factors besides the feedback loops involved. For example, the reward did not improve the performance in the target jump condition but improved the movement time in the target switch condition (though no EMG changes, see below). However, these two conditions had different reward functions, one for minimizing the movement time (MT) and reaction time (RT) but the other for minimizing the deviation from desired movement time.

Now the target switch task, the cursor jump task, and the target jump tasks all reward minimizing movement times. This is also true of the newly designed proprioception-cued reaction time task and the In-Out Target task that we kept from the original study, looking at the short- and long-latency rapid response. This is done to address Potential Confound 2.

Furthermore, movement directions and muscles examined (brachioradialis vs. pectoralis) differ and probably affect the EMG response that is used for quantifying the reward effect.

The new experimental designs now all emphasize lateral triceps activation as the central measure to quantify latencies and feedback gains. This includes the target jump and target switch tasks. This is done to address Potential Confound 1.

Similarly, the cursor jump condition, with a slightly longer latency than the target jump condition but again with a reward function for desired MT, yielded no reward effect either. It makes people wonder whether other task designs would produce the opposite effect on EMG. For example, would the timing and the size of the cursor jump make a difference? What if we reward fast reaction as opposed to maintaining desired movement time in these conditions?

The cursor jump task now rewards fast movement times and emphasizes lateral triceps contraction like all task designs in this study. This is done to address Potential Confound 1 and 2.

The conditions with significant reward effect are mostly those rewarding faster RT and/or MT; the ones rewarding desired movement time generally returns a null effect. The only exception is the target switching condition, which rewards fast MT and shows no reward effect.

The tasks that used to reward desired movement times now all reward fast movement times. This is done to address Potential Confound 2.

However, the target switch perturbation is associated with a peculiar instruction: once perturbed, the participants were required to relax their arms and let the perturbation push them toward the target and stop there. Relaxing while stopping at a target might conflict with the rewarding goal to move fast.

The new experimental design for the target switch experiment now requires moving fast against the perturbation, therefore requiring contraction and no longer relaxation of the main muscle used for analyses. This is done to address Potential Confound 3.

Besides the instruction differences, the conditions drastically differ in muscles examined, movement amplitude/timing and etc. These differences make the conclusions, based on a single specific instantiation of each feedback control paradigm (using the taxonomy by Stephen 2006), debatable.

All experimental tasks now examine the same muscle (triceps lateral head) and are centred on the same shoulder position (a 45 degrees angle), including the visual tasks. Movement amplitudes have been harmonized where possible, with identical perturbation torques across proprioceptive tasks. The displacement amplitude was already identical across visual tasks, and this has been maintained in the new experiments with a visual perturbation. All tasks also now use the same experimental apparatus.

Relatedly, the lack of reward effect in the target switch, cursor jump, and target jump conditions is taken as evidence that feedback responses that rely on sensorimotor and premotor cortices are not modulated by reward, but those relying on prefrontal cortices are. However, it is not clear to me why the LLR condition involves prefrontal associative cortices, but the target jump condition does not. I did not find a discussion of this selective involvement of brain regions either. Given the concern that the specific task designs might cause those null effects, it might be premature to draw this conclusion.

The discussion and conclusions drawn have been re-written to more closely match the new dataset we collected for the new experimental designs. We took particular care to emphasize which statements are speculative and which are not.

The second major concern is whether analyzing a single muscle adequately captures the perturbation effect and the reward effect. For example, the reward improved the performance in the target jump condition (figure 3g), but there is no EMG difference. This has been attributed to other feedback responses that may not be apparent with the task contrast here. But looking at Figure 3J, there is no EMG activity difference between the reward and the control conditions whatsoever. Then, how can the immediate result of EMG, i.e., the movement time, differ between conditions? Is it possible that the muscle activity examined is not relevant enough for the task? This relates to a methodological issue: is the null effect of EMG response to reward caused by the selection of muscles for analysis? For example, the target and cursor jump conditions select the pectoralis muscle only, and thus only leftward target jumps and right cursor jumps are used for analysis. This is reasonable as the pectoralis muscle directly relates to these perturbation directions, but these perturbations probably cause changes in other muscles that are not examined. How can we be assured that any reward effect that is really there is all captured by analyzing the pectoralis only?

The EMG activity in figure 3J is centred on a time window closely matching the perturbation occurrence. There are many ways in which behavioural performance could be improved beyond that window, such as different muscle activation before or after that time window, different contribution from other muscles, better central processing of timing based on the auditory cues, or cocontraction to lock the hand position on the target at the end and finish the movement in a timelier fashion. How that improvement occurs is not the focus of the study, but its presence is a control measurement to ensure that rewarding context does affect the movement. We then analyse the time window matching the feedback response of interest to assess whether that feedback response contributed to the behavioural improvement or not, which is the focus of the current study.

However, we do agree that any study, our own included, would benefit from analysing as many muscles as possible for the sake of completeness. For each experiment, we included the EMG trace of each antagonist muscle to the muscle used for our main analysis, and we quantified and analysed the feedback gains of all muscles we recorded. All these results are consistently included in the

For the questions raised above, I would suggest:

1) Design tasks with similar reward functions, at least.

This is now done.

2) Analyze more muscles.

We now assess feedback gains for all muscles for which we have EMG data: brachioradialis, triceps lateralis, pectoralis major (clavicular head), posterior deltoid, and biceps brachii (short head). We also display the EMG trace of the main antagonist muscle alongside the main muscle of interest for each experiment.

3) Explain why some tasks rely on associative cortices while others on premotor and sensorimotor cortices.

This is no longer relevant to the discussion points and conclusions we make given the new experimental designs and resulting data.

4) Solve the issue of the conflicting instructions for the target switch condition.

This is now done by changing the design of the target switch experiment. Note that the target switch experiment is now re-labelled “target selection” experiment to address another point raised by reviewer 3.

Reviewer #2 (Recommendations for the authors):

It is known that one can obtain a reward, motor performance improves. The authors' aim is to answer the question "which of the nested sensorimotor feedback loops that underly motor performance is/are affected by expected reward.?"

The authors provide a large set of experimental conditions and show that their manipulation of the reward affects the response to some of the perturbations.

A weakness is that the paper lacks a clear hypothesis on how reward would affect the feedback loops. There are several possibilities. It could speed up the information processing, increase the gain, etc. Without a clear hypothesis, it is unclear what the differences are one should be looking for. The authors instead perform a fishing expedition and look for any difference.

We thank the reviewer for bringing up this important point. If fishing expedition refers to an explorative study, as opposed to a confirmatory (hypothesis-driven) study, then indeed the present study would qualify. The dichotomy between explorative and confirmatory work is well formalized (Wagenmakers et al., 2012), with advantages and drawbacks stated and largely discussed for both types of studies. Particularly, omission of explorative work comes with its own bias (e.g., see “The Role of the Hypothetico-Deductive Method in Psychology’s Crisis” section in Scheel et al. (2021)). Importantly, we agree that explorative work should be presented as such (Nosek and Lakens, 2014), and so particular care was put in the present study to ensure this is clear to the reader.

Wagenmakers et al. (2012): An Agenda for Purely Confirmatory Research; DOI:

10.1177%2F1745691612463078

Scheel et al. (2021): Why Hypothesis Testers Should Spend Less Time Testing Hypotheses; DOI: 10.1177/1745691620966795

Nosek and Lakens, 2014: Registered Reports A Method to Increase the Credibility of Published Results; DOI: 10.1027/1864-9335/a000192

A second weakness is that the conditions differ not only in the aspect that is presented as the reason for performing the task but also in several additional aspects. For instance, the paper contains two reaction time tasks. One is based on visual input, the other on proprioceptive input. However, the visual one is also a choice reaction time, whereas the proprioceptive one is not. The most serious variation is that what the authors reward differs between the experiments. For instance, performance in the target-switch condition is rewarded for short movement times whereas small deviations from the desired movement time are rewarded in the target-jump condition. In other conditions, the reward is based on time-in-target. So, any difference between the experiments might be due to this difference in reward criterion.

All reward functions now reward minimization of movement time and so have the same reward criterion. Note that the task measuring the short- and long-latency rapid response still rewards time in target instead of movement times directly, but this is strictly equivalent to rewarding short movement times in practice because trial duration is fixed in that experiment, i.e., only the mathematical formulation differs. This is done to address Potential Confound 2.

A third weakness is that the authors use 'feedback' for aspects of control that are feedforward. Feedback control refers to the use of sensory information to guide the effector to the target. However, switching to another target (second experiment) is a decision process (selecting the goal), and is not related to reaching the target. It is unclear how this relates to the "target jump" condition, which can be interpreted as resulting from feedback control.

Generally, this points to another discussion of when feedforward control stops, and feedback control starts. A proposed view, which we favour, is that feedback loops do not act as “sheathed” system with well-isolated ascending and descending loops, but as a nested set of loops which can bypass and override each other, while receiving constant streams of top-down modulation as well (Scott, 2016, Reschechtko and Pruszynski, 2020, particularly the last section). In this context it is reasonable to consider a decision-making process as being part of a feedback loop, similar to previous empirical work (Nashed et al., 2014, especially experiment 4; De Comite et al., 2021, DOI:10.1101/2021.07.25.453678).

We agree that this leans toward a broader definition of what feedback control is. Our main consideration is that this is a complex question that steers away from the purpose and scope of this study, although we concur that this is a very interesting and relevant question for the field.

Note: the studies cited above are referenced in the main manuscript, unless a DOI was provided.

A fourth weakness is that the analysis (or the written report of it) is sometimes confusing. For instance, the authors use terminology R1, R2, R3 as defined by Pruszynski et al. (2008). They don't report the definitions themselves (e.g.: R2 corresponds to 45-75 ms after the perturbation). Despite explicitly citing this paper, they don't seem to use these definitions. Instead, they redefine them as "R2 and R3 epochs were taken as the first 25 ms.… after each participant's LLR latency". By using this flexible definition of epochs, the epoch is not an independent variable anymore.

We edited the methods and Results sections throughout to be clearer in our description of the experimental design and analyses.

Regarding the specific point raised up, the use of an epoch definition that is fixed in time has its own limit. For instance, one would not expect the LLR response to arise at 45 ms both for an individual with a short arm length and one with a long arm length, simply due to differences in transmission delays. Therefore, not adjusting the time window accordingly will bias the results in a way that is unnecessary if we have knowledge of response latencies.

More critically, latencies themselves may (and in some experiments do) vary with a rewarding context, which is the manipulated variable in our experimental designs. Therefore, by not adjusting for that variation, we are in fact using window positions (relative to the onset of the feedback response of interest) that will vary as reward varies. That would make the epoch boundaries dependent on – not independent from – the presence or absence of reward.

A fifth weakness is that it is unclear in the SL/LL experiment whether the stimulus (the stretch of the muscle) is affected by the reward, as the mechanical stiffness of the muscle might have been affected by the expected reward (e.g. by co-contraction).

We have included average EMG activity at baseline (350 to 300 ms before the perturbation occurs) for all experiment, both for the main muscle used and its antagonist. Considering a follow-up comment made below, it appears we have missed some information on how we calculated trial-bytrial EMG baselines and therefore what they represent. A paragraph has been added on that point to section 4.5 of the new manuscript.

Perhaps a more direct way of measuring mechanical stiffness is by looking at maximum excursion in the kinematics following the mechanical displacement, which we display in the figure below. However, a clear difference was not consistently observed across participants between rewarded and non-rewarded trials in the LLR condition.

**Author response image 1.** Position of maximum excursion following the perturbation in the condition with an inward (counterclockwise) push and an outward (clockwise) target. Trials where reward was provided are colorcoded in green, and trials where no reward was provided are color-coded in red. The triangle indicates the starting position from which the perturbation occurred. Each panel represents one participant (N=16).

There are at the moment some conflicting views on the relationship between reward and motor variability and learning. Well-designed experiments would be able to help to advance the field here. As the authors varied much more between the experiments than only the loop involved, they have not convinced me that the differences they report are indeed related to differences in how expected reward affects the nested sensorimotor feedback loops.

Using page numbers would have facilitated feedback

This is now done.

Title: In my understanding, it should contain "expected" rewards, as the responses that are investigated occur before the reward is provided.

"a 10 cm target appeared at 20 degrees" Use comparable units (all degrees or all cm) and clarify whether the size is radius or diameter.

We now specify that the sizes indicate radii in the methods.

The degrees unit refer to the angle formed between the arm and the forearm (see Figure 2a of the new manuscript). Therefore, a target in degrees would not have the same size in cm for a participant with a long forearm compared to one with a short forearm due to angular projection. Consequently, degrees and cm are not strictly convertible units here, and we have now specified this in the text to ensure this is clear to the reader (section 4.4.1).

A figure explaining the time-course of a trial might be helpful.

"an inward" Better use "(counter-)clockwise"

In several captions it is mentioned: "The left panels show EMG at trial baseline (see methods)", but in the methods, there is no mention of "trial baseline". There is mention of a "mean baseline scalar" and that "EMG measures during the task were then normalised by each muscle's baseline scalar." I have no idea what this means. Is the scalar subtracted, expressed in multiples of baseline activity? And are the plotted signals those after normalising?

We thank the reviewer for pointing out the missing information. We have added this paragraph to section 4.5 in the methods:

“For all experimental designs, trial-by-trial baseline EMG activity was measured in a 50 ms window from 350 to 300 ms before displacement onset, while participants were at rest, background loads were applied, and after targets and reward context were provided. For the target Jump and Cursor Jump tasks, this was measured in a 50 ms window from 350 to 300 ms before target appearance instead of before displacement onset because movements were self-initiated, and displacements occurred during the movement. However, the same target was displayed in every condition at the start of a given trial in those two experimental paradigms. Note that these trial-by-trial baseline EMG signals are distinct from the 4 baseline trials described above in this section, which were done before the task started and were used for normalization of EMG signals. The trial-by-trial baseline EMG signals were not used for EMG normalization.”

Additionally, we have specified where in the task design the trial-by-trial baselines are positioned where appropriate, in sections 4.4.2 to 4.4.5.

It is unclear how latencies and reaction times are determined. There are many options for this, and the results of the analyses of latencies depend critically on which options are chosen.

We agree that the method used to obtain latencies and reaction times are particularly important. For that reason, we used previously employed methods that were successfully employed in that context, specifically Pruszynski et al. (2008) for the reaction times and Weiler et al. (2015) for the other experiments. Weiler et al. (2015) is particularly interesting in that regard, as it dedicates a significant portion of the methods describing, explaining, and analysing the Receiver Operating Characteristic (ROC) method we employed to estimate latencies in most tasks (reaction times excluded). We followed the procedure as strictly as possible, and detail where appropriate the parameters we used when they were different (see below).

Regarding reaction times, section 2.5. in the results indicate:

“Reaction times were defined as when the (processed) triceps EMG signal rose 5 standard deviations above baseline level (Pruszynski et al., 2008) for 5 ms in a row (Figure 5d).”

Regarding latencies, section 4.6 in the methods specifies:

“To determine the time at which EMG signals for different task conditions diverged, we used Receiver operating characteristic (ROC) analysis. We used the same approach as in Weiler et al. (2015), using a 25-75% threshold of area under the curve (AUC) for establishing signal discrimination. The threshold was considered reached if two consecutive samples were greater than the threshold value. Discrimination was done for each participant and each reward condition independently, using all trials available for each contrast without averaging. Once the AUC threshold was crossed, we performed a segmented linear regression on the AUC before it crossed the 25-75% threshold. We minimized the sums-of-squared residuals to find the inflexion point, that is, where the two segments of the segmented linear regression form an angle (see Weiler et al. (2015) and analysis code online for details).”

Particularly, the analysis code for the segmented linear fitting is freely available online at the URL:

https://journals.physiology.org/doi/suppl/10.1152/jn.00702.2015

"Conversely, the LLR arose.… (LLR not occurring, Figure 2c)." This is not my interpretation. In both cases, an LLR is present, in one case much stronger than in the other. Secondly, the effect of the task is not present at the onset of the LLR, but starts at a moment the LLR has already started. The authors refer to this latter time as the latency, but the figure shows a clear SL and the onset of the LL, which is clearly before the effect kicks in.

We agree that this is a misleading statement, as LLRs can occur in both conditions, although in different strengths. Overall, the section considered is largely re-written, so this does not apply directly anymore, but we avoided the “LLR not occurring” phrasing in the new text.

Figure 2: explain graphically what continuous and dashed lines signify, and green/purple. I can't follow panel d: In my understanding, SLR and LLR are determined by subtracting data from within the same experiment in a different way. How can this have (for at least one participant) such a large effect on the difference of time-on-target between rewarded and non-rewarded trials? How do the data in panel f link to those in panel e?

We have added a graphical legends to the EMG panels indicating the color-code. Additionally, we now indicate in panel A of each figure which condition is the control and which is the manipulation. This (panel A) is also referred to in the caption for the EMG signal panels. Finally, we added a visualisation of kinematics for each experiment to better showcase the comparison made.

"For this analysis, we did not use a log-ratio" This is not clear to me. You normalised the EMG and expect a change in gain. So why not a log(ratio)?

EMG signal strength varies over time, so normalization across two different time windows would be different. This is not a problem in the first analysis (figure 2e in the original manuscript, 3f in the new manuscript) because we compare a time window in condition 1 to the same time window in condition 2. However, in the second analysis across epochs, this is a problem (figure 2f in the original manuscript, 3g in the new manuscript). We specify in the text for clarity:

“For this analysis, we did not use a log-ratio, because EMG activity was not scaled similarly in R2 and R3 epochs (Figure 3d-e), and that difference would lead to a mismatched ratio normalisation across epoch, hindering comparisons.”

It would help if all the figures were showing similar data in the same format. The various ways to plot data are confusing.

The figures now follow a common layout across all experiments.

Please add plots of the displacement as a function of time. Specify in caption whether the EMG plots show the means of all participants or of a typical single participant

We are unsure of what is referred to by “the displacement as a function of time”. If this refers to the movement trajectories, they are now added for all experiments, both for one participant and the average trajectory of all participants. The caption in all relevant figures now specify “average triceps EMG signal across participants” (new segment underlined).

Please make sure that all major aspects of the task are mentioned in the results text. Now the most essential information (what is rewarded in each experiment) is missing, whereas total irrelevant details (that no-reward corresponded to 0 ¢ CAD) are provided. Additionally, understanding why mechanical perturbations are provided as torques (and not as displacements) might be easier to follow if you briefly mention in the Results section that an exoskeleton is used.

Figure 1a is very useful to help the reader to understand the authors' line of thought. Unfortunately, the authors don't lead the reader through this figure. As latencies relate to the hierarchy, it might be simpler to add the various loops from panel b to panel a, and remove panel b.

We agree panel B is superfluous and potentially distracting. We have removed it from the manuscript.

"Codol et al. (2020)." Is it 2020a or 2020b?

We thank the reviewer for spotting this missing information. It is 2020a. This is now added to the text.

I am not sure where (Dimitriou et al. 2013) claimed that responses to a cursor jump have a longer latency than to a target jump (section "Online visual control of limb position was also unaltered by reward"). In an earlier study, the opposite has reported (Brenner and Smeets 2003), which is in line with the scheme in figure 1a.

In light of the comment above, it appears our phrasing was ambiguous. The sentence should have read (new element underlined):

“Next, we assessed feedback response due to a cursor jump rather than a target jump. This feedback response is sensitive to position of the limb like the LLR, but it displays longer latencies than the LLR due to relying on the visual sensory domain (Dimitriou et al., 2013).”

However, this sentence was removed following editing of the manuscript’s Results section, and therefore this does not apply directly anymore.

What are 'goal-sensitive' feedback responses? What is 'absolute response latency'? These concepts are not explained.

This does not apply directly anymore, as we have re-written large parts of the manuscript and have removed those terms from the main text to improve clarity.

Please be consistent. The authors use, 'stretch reflex" and "short-latency reflex" interchangeably. In the abstract and discussion, the authors refer to "eight different kinds of sensorimotor feedback responses". In figures 1a and 6a, I count nine kinds of responses. What happened to the ninth one? In table 1, I count 5 tasks. Please provide a mapping from tasks to responses. Secondly, provide for all experiments similar plots. Now the exoskeleton that is very relevant is not drawn, but the endpoint-Kinarm that is not essential is drawn.

We have harmonized the use of “stretch reflex” to “rapid response” and “short-latency reflex” to “short-latency rapid response” everywhere in the text. This wording choice is also motivated by other comments from reviewer 3. We now provide a table indicating the source of each feedback response represented in figure 8 of the new manuscript. Each feedback response now has their own dedicated figure (excepted the choice reaction time task), and all figures now have a similar layout to facilitate reading.

Discussion: this section contains an aspect that could have been discussed in the introduction (cortico-cerebellar loops not assessed), as this is not related to the results or conclusions. I miss a discussion of how behaviour can be improved by expected reward with such little changes in the underlying sensorimotor control. A last item that could be discussed is that reward might affect behaviour not only by expected reward but also through a learning mechanism, so the (lack of) reward will affect the trial after the (lack of) reward.

The discussion is now completely re-written.

Reviewer #3 (Recommendations for the authors):

The question on how reward or value impacts feedback processes is important to understand. Previous studies highlight how reward impacts motor function. Given feedback is an important aspect of motor control, it is useful to know which feedback responses may be specifically impacted or altered by reward.

A clear strength of the manuscript is the systematic evaluation of different feedback processes reflecting different latencies and behavioural contexts to initiate a response. These differences reflect differences in the underlying neural circuitry involved in each of these feedback processes. Examination on how reward impacts each of these processes using similar techniques and approach provides a comprehensive examination on how reward impact feedback responses and a much cleaner overview of the problem, rather than a fractured examination if explored over many separate studies.

The manuscript uses a functional taxonomy suggested by Scott (2016) to define the behavioural contexts examined in the paper. In most cases, the experimental paradigms match these taxonomies. However, some confusion seems to occur for responses elicited at ~50ms following mechanical disturbances which includes two distinct types: 1) goal-directed online control and 2) triggered reactions. These two conditions are behaviourally quite different as the former maintains the same goal before and after the disturbance, whereas the latter switches the behavioural goal, and thus, feedback responses are now set to a new spatial goal. Triggered reactions are examined in the present study, but it is assumed that this reflects goal-directed online control (the former). Thus, responses at ~50ms can reflect substantially different behavioural conditions (and likely processes) and these distinctions should be recognized.

I think the simplest approach for quantifying the impact of reward on corrective responses is to compare corrective responses in a single condition with and without reward. However, the manuscript used paired behavoural conditions to look at the difference in EMG between contexts and then identify if this EMG difference changes between rewarded and non-rewarded trials. This makes the material more complex to understand and follow. Admittedly, the use of this EMG difference between conditions works well if reward should increase a response for one context and decrease it in the other. For example, target jumps to the left compared to the right increase pectoralis activity for the leftward jump and decrease for the right jump. Reward should enhance both of these reciprocal responses (increase the first and/or decrease the latter) and thus lead to a larger EMG difference for rewarded trials. So this contrast approach makes sense in that experiment. However, the contrast for goal-tracking (actually should be called goal-switching) experiment contrasts the switching goal condition with a control condition in which corrective responses were generated to the original spatial goal. In this situation, both contexts could show an increase in EMG with reward, and in fact, that appears to be the case shown in Figure 3e (top panel shows both conditions have a slight increase in EMG for rewarded trials). However, by looking at the difference in EMG between conditions, this reward-related activity is removed. I think these two behavioural contexts should be assessed separately.

We have completely re-designed and re-collected the experiment relating to target switching so that it more closely matches the task used for the SLR and LLR measurements. We believe the new task design should alleviate the concern raised here. This was done also to address Potential Confound 3 and 4.

Critically, the baseline condition where corrective responses were generated to the original goal fills the void regarding goal-directed online control mentioned in the previous paragraph that occurs at ~50ms. If there is a significant change in EMG for the goal-directed online control, then it could be used as a contrast for the target switching task to address whether there is any greater increase in EMG response specifically related to target switching.

I think there is some confusion with regards to some of the functional taxonomy and the experimental paradigms to assess these processes from the functional taxonomy outlined in Scott (2016). Specifically, there are two distinct behavioural processes that occur at ~50ms related to proprioceptive disturbances: there is 1) online control to an ongoing motor action where the behavioural goal remains constant, and 2) triggered reactions to attain a new goal. The present study is the latter and was developed by Pruszynski et al., 2008 (should be cited when describing the experiment) and is really just a spatial version of the classic resist/don't resist paradigm. However, this Target In/Out task is assumed to be the former both in Figure 1 and the text. These are distinct processes as the goal remains constant in the former and switches in the latter. The former is comparable to a cursor jump task where the arm (or cursor) is shifted, but the goal remains the same. I think Figure 1 and the text needs to recognize this distinction as outlined in Scott (2016).

We agree that the LLR contains several responses, including one that relates to online control of the limb (earlier) and one that relates to goal switching (later). As we use figure 1 as the basis for figure 8 to provide a “graphical abstract” of the set of results we obtain, we are trying to keep an approach based on task used. In light of the comment above it appears that this could be improved. Specifically:

- The “target switch” task is renamed “target selection” task in figure 1 and 8 and in the main text
- The new “target selection” task and the “alternative target” task now correspond to an “action selection” function.
- We indicate that the first two contrasts are part of the in-out target experiment
- Each response (SLR and LLR) relates to a different contrast within the in-out target experiment
- The short-latency contrast now correspond to the “online control of the limb” function in figure 1.
- The long-latency rapid response functionally relates to online goal switching

We also move away from the “stretch reflex” nomenclature and instead use the original “rapid response” nomenclature as in Pruszynski et al. (2008).

Finally, the main text now refers to the original study when introducing the task used for the LLRSLR.

“This yielded a 2x2 factorial design, in which an inward or outward perturbation was associated with an inward or outward target (Pruszynski et al., 2008).”

This distinction between triggered reactions and online control is important as triggered reactions are related to another task examined in this study, proprioception-cued reaction time task. These are essentially the same tasks (disturbance drives onset of next action in Scott 2016), as the only difference between them is the size of the disturbance with triggered reactions using large disturbances leading to responses at ~50ms and small disturbances for reaction time tasks leading to responses starting at ~110ms. These are likely not distinct phenomena, but a continuum with the latency and magnitude likely impacted by the size of the mechanical disturbance, although I don't think it has ever been systematically examined. Critically, they are very similar from a behavioural context perspective. Interestingly, the present study found that reward shortened the latency and increased the magnitude for the proprioceptively cued reaction time task and increased the gain for the triggered reaction, but not the latency likely due to the fact the latter hit transmission limits. The manuscript should recognize the commonality in these behavioural tasks when introducing the tasks. Perhaps these experiments should be grouped together. I think the strategy of the manuscript was to introduce experiments based on their latency, but this creates a bit of an artificial separation for these two tasks.

We thank the reviewer for raising this very interesting point. We have added it to the main text, in the new discussion in the paragraph where we discuss transmission delays, as it is particularly relevant there:

[…] “Consequently, the LLR has little room for latency improvements beyond transmission delays. This is well illustrated in the proprioception-cued reaction time task, which holds similarities with the task used to quantify the LLR response but with a smaller mechanical perturbation. Despite this similarity, latencies were reduced in the proprioception-cued reaction time task, possibly because the physiological lower limit of transmission delays is much below typical reaction times.”

It would be useful to add a goal-directed online control experiment to assess EMG responses when reaching to spatial goals with mechanical disturbances with and without reward. This would provide a nice parallel to the experiments examining cursor jumps to explore online control of the limb. Previous work has shown that increases in urgency to respond to a postural perturbation task led to increases in long-latency responses (Crevecouer et al., JNP 2013). Urgency in that study and reward in the present study are related as reward was based on how long individuals remained at the end target which is similar to the need to return faster to the target in Crevecouer et al. There may be specific differences between posture and reaching, but the basic principle of corrective responses to attain or maintain a goal are similar. In fact, you second experiment incorporates a simple goal-directed online control task with mechanical disturbances in the goal-tracking task displayed in 3a. This could be analyzed on its own to fill this void.

As the experimental design of the second experiment is now different and we have re-worked the nomenclature in figure 1, this point may not apply directly anymore. We look forward to discussing it further should it be needed.

The experimental paradigms use comparisons between two conditions (termed a control and a manipulation condition, in some cases). I'm not entirely sure why they did this as a simpler strategy would be to examine the differences between rewarded and unrewarded trials for a given condition. The logic (and value) may be that multiple feedback processes could be impacted by reward and you wanted to see the incremental change between feedback processes. However, looking at the difference in EMG in the goal-tracking task makes me wonder if the authors missed something. It looks like both the control and manipulation condition show a slight increase in EMG in Figure 3e. However, the statistical test simply looks at the difference in these responses between control and manipulation, and since both show a slight increase for rewarded trials, the difference in EMG removes that increase observed in both signals resulting in no difference between rewarded and non-rewarded trials. I think the control and manipulation conditions should be separated as I don't think they are directly comparable. While lines overlap in the top panel of Figure 3e, it looks like the rewarded trials for the target switch condition may show a reduction in time and an increase in magnitude during the LLR for rewarded trials (the challenges of a dashed line).

The online control condition from the target switching task (Figure 3a) could be examined on its own. If a contrast design was important, that would require pairing the resistive load with an assistive load, or perhaps loads to deviate hand orthogonal to the target direction to parallel the cursor jump experiment.

We have completely re-designed and re-collected the experiment relating to target switching so that it more closely matches the task used for the SLR and LLR measurements. We believe the new task design should alleviate the concern raised here as one would now expect a rewarding context to increase the response in each of the diverging conditions. This was done also to address Potential Confound 3 and 4.

I think it's confusing to frame the first two experiments based on short-latency, long-latency, and slower long-latency. You have provided a clean approach to introduce different feedback processes based on behavioural features of a task (Figure 1). I think you should stick to these behavioural features when describing the experiments and not talk about short-latency, long-latency and slower long-latency when developing the problem and experiments as this confuses the taxonomy based on behaviour with time epochs. As you carefully point out, there are many different layers of feedback processing and so epochs of time may include influences from many pathways and two processes may happen to take the same time (i.e. goal-directed online control and triggered reactions). Further, there are two distinct processes at ~50ms which needs to be clarified and kept distinct. Thus, behavioural context and not time epoch is important to maintain. This is why the later experiments on cursor jump, target jump and choice reaction time are much easier to follow.

We have reframed those two experiments and now stay closer to the task-related and behavioural features we measure, as suggested in this point. This is particularly apparent in the new format for figure 1 and 8 and in the discussion.

The impact on reward on baseline motor performance is a bit confusing. It is not clear that various statistics of motor performance in the various conditions is related to the baseline movements without disturbances or with disturbances. This may be stated in the methods but should be clearly stated in the main text and legends to avoid confusion.

When we refer to the motor performance (MTs) in the result, we have added a sentence describing in more details the method used. For instance, in section 2.1 (new sentence underlined):

“Before comparing the impact of rewarding context on feedback responses, we tested whether behavioural performance improved with reward by comparing movement times (MT) expressed. To do so, we computed for each participant the median MT of trials corresponding to the conditions of interest (Figure 2a, rightmost panel) for rewarded and non-rewarded trials and compared them using a Wilcoxon rank-sum test. Indeed, median MTs were faster during rewarded trials than in nonrewarded ones (W=136, r=1, p=4.38e-4, Figure 2c).”

Generally, for each task, we took the conditions of interest represented in panel A of each figure and calculated the median MT for each participant in those conditions, for rewarded and non-rewarded trials. This resulted in two median MT per participant (rewarded, non-rewarded). We computed a Wilcoxon rank-sum on these pairs of median MT to assess statistical significance.

In the new task designs used, all conditions of interest include a perturbation, so there is always presence of a disturbance in the trials included in calculation of median MT. Note that this was also true in the previous experimental designs, although this is no longer relevant to the current version of the manuscript.

I don't think it is useful to talk about goal-tracking responses for experiment 2 as the term tracking is usually reserved for moving targets. I kept re-reading trying to understand how the goal was moving in the task and how the individual was tracking it, but this clearly didn't occur in this experiment! Rather, this task is probably best characterized as goal switching (as stated in methods section). The term slower in the title is also unnecessary and confusing. Again, stick to behavioural features of the task, not time epochs.

We agree with this point and have re-designed figure 1 and 8 accordingly. This task is now renamed “target selection”, which we think is more accurate, as no switch occurs in the new experimental design that we use for assessing that feedback response. This is also categorized functionally as an “action selection” process. This change has also been done throughout the text.

[Editors’ note: what follows is the authors’ response to the second round of review.]

The manuscript has been improved but there are some remaining issues that need to be addressed, as outlined below:

All reviewers find this paper (or a revised version of the previous submission) a timely effort to study how the motivational factor affects motor control. The methodological improvements are also helpful in addressing previous concerns about consistency across experimental tasks. However, the reviewers converged on two major concerns:

1) The link between functional tasks and their assumed feedback loops is poorly specified. For example, goal-switching and online action selection appear the same in function. Target jump and cursor jump are also similar. The LLR contrast and the target selection yielded similar latency and had the same sensory input but were treated as involving different feedback loops. The functional taxonomy proposed in Scott 216 paper was not meant to suggest that each functional process was supported by a different "feedback loop." Thus, we suggest that the introduction should be less conclusive about the functions that the various experimental paradigms address and whether they tap into different or the same feedback loops. Perhaps simply referring to the existence of these paradigms in the introduction is enough for this descriptive research. At the same time, the link between functional tasks and their assumed feedback loops should be discussed more appropriately in the discussion.

Our interpretation is identical to the one described above, that is, that each functional process is not supported by a different "feedback loop.". First, we draw a distinction between a feedback loop and a feedback response. A feedback loop will have a biological meaning (e.g., the spinal circuitry), while a feedback response is simply the behavioural outcome following a perturbation. For instance, the circuitry that produces the Long Latency Response (LLR) will also contribute to the response observed in the Target Selection task. A feedback loop will likely contribute to movements for which there is no externally applied perturbation as well and thus for which there is no feedback response. Therefore, the perturbation is merely a means to force a response whose properties can be used to infer about the nature of the feedback loop(s) involved.

Second, even if we were to replace feedback “loop” by feedback “response in the statement above (“each functional process is not supported by a different "feedback response"), this would still be misleading. A feedback response is sensitive to a specific goal by virtue of the information integrated in the circuitry implementing it, but the response does not exist “by design” to implement that function, as the term “is supported by” implies.

Considering the comment above, this view is not clear enough from the manuscript as it stands. To better convey this viewpoint, we have amended the second paragraph of the introduction. Specifically, we changed “each feedback loop is governed by different objectives” to “each feedback loop is sensitive to different objectives”, and we added the following statement:

“Here, the term “feedback response” refers to a behavioural response to an externally applied perturbation. The term “feedback loop” refers to a neuroanatomical circuit implementing a specific feedback control mechanism which will lead to all or part of a behavioural feedback response.”

We have also expanded on how each the feedback response of each experiment relates to the functions of figure 1 as each experiment was introduced in the results.

“Section 2: The SLR corrects the limb position against mechanical perturbations regardless of task information, whereas the LLR integrates goal-dependent information into its correction following a mechanical perturbation (Pruszynski et al., 2014; Weiler et al., 2015).”

“Section 2.3: Therefore, the divergence point between each condition is the earliest behavioural evidence of a target being selected and committed to.”

“Section 2.4: While this experimental design is similar to the LLR contrast used in the In-Out Target experiment, a key distinction differentiates them. In the proprioception-cued reaction time task, the movement to perform can be anticipated and prepared for, while for the LLR the movement to perform depended on the direction of perturbation, which is unknow until the movement starts. Specifically, in the LLR, a perturbation toward or away from the target requires a stopping action to avoid overshooting or a counteraction to overcome the perturbation and reach the target, respectively. Therefore, the behaviour we are assessing in the reaction time task represents an initiation of a prepared action, rather than an online goal-dependent correction like the LLR.”

“Section 2.5: In a new task using the same apparatus (Figure 6a), a visual jump of the cursor (indicating hand position) occurred halfway through the movement, that is, when the shoulder angle was at 45 degrees like the Target Selection task (Figure 6b). This allowed us to assess the visuomotor corrective response to a change in perceived limb position (Dimitriou et al., 2013).”

Finally, we have amended the relevant Discussion section (Section 3.3) according to individual reviewer comments, as described in details below.

2）The critical methodological problems that might affect the validity of the findings should be addressed. These problems include how the latency is determined (e.g., the need to have other methods to confirm the latency estimation, the need to have a fixed time window), which segment of EMG should be analyzed (e.g., R2 and R3 splitting), and which muscles should be used for analysis (e.g., most analyses based on one muscle though all muscles are monitored; having an arbitrary choice of muscles is a warning sign for p-hacking). These problems are more detailed in Reviewer 3's comments.

Regarding the validity of the metrics used, in the individual responses below:

- We demonstrate that the effect of reward on feedback gains remains similar if using a fixed window.
- We demonstrate that the latency estimation method we use in this study is superior to alternative choices. Specifically, we performed a series of simulations on the four alternative latency estimation methods suggested as well as the one we used and show that the other methods do not perform as well in the context of our dataset. We then proceed to show that the EMG signals on which latency estimation is done in this study likely express amounts of noise that are greater than what alternative methods can handle. Finally, we illustrate this on data from individual participants.

In light of comments made by the reviewers, we have removed the R1-R2-R3 terminology in this manuscript, as it does not correspond to the canonical way this terminology is used in the main literature. The comparison between R2 and R3 was also removed, as it distracts away from the main points of this study in its current form.

Regarding p-hacking, there are in fact several layers of protection against it in the current study. Here we are quoting elements of one of our answers further below:

Quote starts

[For any given task,] if a muscle’s activity does not diverge between conditions, one cannot estimate the latency of that (non-existent) divergence. For our study, this situation would indicate that there is no relation between the geometry of the task (orientation of movement) and the geometry of the muscle (orientation of pull) considered. Since there is little redundancy in the geometry of the muscles we recorded from (and in the musculoskeletal system overall), it is essentially impossible to find an experimental design that would result in two muscles diverging for the same movement, forcing one to decide pre-collection which muscle to emphasize. Here, we decided to emphasize the triceps in all our experiments to remain consistent with the experiment measuring the SLR and LLR, since this is the experiment that the reviewers had found most convincing and appealing in the first round of review.

We could have designed the experiments to emphasize another muscle (i.e., which muscle we want to emphasize is arbitrary). But having only one muscle whose EMG signal diverges between conditions prevents us from exploring all the muscle signals post-hoc and picking whichever would be the most convenient. In other words, since the muscle to analyse must be declared pre-collection, one cannot decide on post-hoc changes once data is collected. That situation is in fact robust to p-hacking rather than prone to it.

In addition, since we use the same muscle across experiment for estimating latencies, there is no rationale for changing post-hoc the muscle considered for one experiment without having to change it for all five experiments. And since the first experiment (assessing the SLR and LLR) is preserved from the first version of this draft, this acts as an anchor point preventing unchecked data mining with respect to which muscle to use for latency estimation.

More generally, we would like to point out that by re-designing and re-collecting four out of five experiments, the original (first) draft of this study acts as a check against data mining. The scientific question at hand, the variables of interest (feedback gain and latency), the analysis to estimate them (difference of integrals over a time window, ROC analysis), and statistical tests used (Mann-Whitney rank-sum tests) are all strictly the same as in the first draft.

Quote ends

More generally, many comments made throughout this review suggest that we are not explaining the experimental designs and their motivation clearly enough in the main text. We have included two additional paragraphs in section 2.1 to better describe the experimental process, alongside additional panels in each figure breaking down the experimental design at hand.

Note that for this reason, the panel letters that the reviewer indicate in their comments below will not match those in the new manuscript. For convenience, we specify the new panel labels where appropriate in the responses below.

Reviewer #1 (Recommendations for the authors):

The study aims to test whether sensorimotor feedback control is sensitive to motivational factors by testing a series of tasks that presumably rely on different feedback loops. The first strength of the study is that all the feedback control tasks were designed with a unified upper-limb planar movement setting with various confounding factors under control. Its previous submission a year ago had received some major criticisms, mostly about inconsistency across tasks in task goals, analyzed muscles, and reward functions. The new submission has used re-designed experiments to keep consistency across tasks and successfully addressed most, if not all, previous major concerns. As a result, this study gives a more complete picture of how motivation affects feedback control than previous studies that did not scrutinize the feedback loop involved in the task.

The study found that the fastest feedback loops, both for visual and proprioceptive feedback, are free from the effect of reward in terms of muscle response. The earliest reward-sensitive feedback loop has a latency of about 50ms, depicted by the response to the proprioceptive perturbation. Reduced response latency and increased feedback gains underlie the reward-elicited improvements, but their roles vary across tasks.

The weakness of the study is that the underlying mechanisms for the heterogenous results are speculative. Though the study included five tasks and one previous dataset, it did not conduct experiments for some tasks, or failed to have electromyography measurements. These tasks include those related to vision-cued reaction time, alternative targets, and choice RT. The incomplete task set might prevent drawing conclusive explanations for the current findings. The theoretical account to explain the increased feedback gain is so-called anticipatory pre-modulation, but this term is unspecified in any detail based on the present findings. Using this account to explain the finding that the cursor jump task (in contrast to the target jump) failed to induce a reward effect in feedback gain, the authors hypothesize that the anticipatory pre-modulation does not work for the cursor jump task since it cannot prime the participants with the probability of a cursor jump. I find this explanation unsatisfactory: the probability of the jump to either direction is similar for both the target jump and cursor jump tasks as they share identical trial designs.

We thank the reviewer for pointing out the lack of a clear definition on the “anticipatory premodulation” term used here. We have amended the discussion to introduce a definition of the term where first introduced, in section 3.2:

“Unlike transmission delays, the strength of a feedback responses can be modulated before movement occurrence, that is, during motor planning (de Graaf et al., 2009; Selen et al., 2012), which we will refer to as anticipatory pre-modulation here.”

And a more detailed description of what form we expect this mechanism to take at the neural level:

“In a general sense, pre-modulation results from preparatory activity, which at the neural level is a change in neural activity that will impact the upcoming movement without producing overt motor activity – that is, output-null neural activity (Churchland et al., 2006; Elsayed et al., 2016; Vyas et al., 2020). Regarding feedback gain pre-modulation, this means that in the region(s) involved there is an output-null neural activity subspace from which the neural trajectory unfolding from the upcoming movement will respond differently to a perturbation. Importantly, not all preparatory activity will yield a modulation of feedback gain, or even task-dependent modulation at all. An extreme example of this distinction is the spinal circuitry, where preparatory activity is observed but does not necessarily translate into task-dependent modulation (Prut and Fetz, 1999). This is also consistent with our result, as we observe no change in feedback gain with reward in the SLR. Therefore, since not all preparatory activity is equivalent, we do not propose that the presence of any preparatory activity, even task-related, will automatically result in reward-driven modulation of feedback gains.”

Regarding the last point, we agree that there is no difference in jump probability between the cursor jump and target jump designs. The reviewer’s point highlighted that the main text may be confusing. We had initially written:

“Therefore, one could consider a version of these tasks in which participants are primed before the trial of the probability of a jump in either direction (Beckley et al., 1991; Selen et al., 2012). In the Target Jump task, the above proposal predicts this should pre-modulate the gain of the feedback response according to the probability value. In the Cursor Jump task it should not.”

We edited this segment to now read (section 3.2, third paragraph):

“Therefore, one could consider a probabilistic version of these tasks in which the probability of a jump in each direction is manipulated on a trial-by-trial basis, and participants are informed before each trial of the associated probability (Beckley et al., 1991; Selen et al., 2012). Previous work shows that this manipulation successfully modulates the LLR feedback gain of the upcoming trial (Beckley et al., 1991). Given our hypothesis, it should premodulate the feedback gain following a target jump, but not following a cursor jump, because the absence of reward-driven feedback gain modulation would indicate the circuitry involved is not susceptible to anticipatory pre-modulation.”

In sum, the study achieved its goal of testing whether the motivation factor improves feedback control when different feedback loops are predominantly involved in various tasks. The experimental tasks are carefully designed to avoid multiple confounding factors, the analysis is solid, and most of the results are convincing (with the exception of the "significant" difference in Figure 5f). The study aim is more explorative than hypothesis-driven, thus limiting the insights we can obtain from the heterogeneous results. However, through systematically studying feedback loops in a unified experimental framework, the study provides more insights into the effect of motivation on sensorimotor feedback control in the aspect of response latency and gain and thus can serve as a new stepping stone for further investigations.

Labeling the experiments with numbers would be good, especially considering the paper also includes an online dataset (Figure 5g and 5h).

We considered labelling the experiments using numbers or even letters, but ultimately avoided it because it would assign arbitrary numbers to each experiment without semantic meaning attached to it, rendering the reading experience less explicit and thus more tedious.

Instead, we decided to actively name experiments with an explicit reference to their content (e.g., the cursor jump experiment is the experiment in which the cursor jumps halfway through the reach) to avoid forcing the reader to hold into memory a mental label-to-content “map”.

Page 5: "Next, we assessed the time of divergence of each participant's EMG activity between the reward and no-reward conditions using a Receiver Operating Characteristic (ROC) signal discrimination method…"

Does this refer to the divergence of EMG activity from the baseline, not between the two conditions?

It refers to the divergence between the inward and outward perturbations conditions (see new figure 2b-d). We thank the reviewer for spotting out this typo.

Figure 2b: what do the two colors mean? Reward and no reward?

The colors refer to the two conditions that we contrast to show and characterize the SLR, in this specific case, trials with an inward perturbation and those with an outward perturbation. A legend has been added to the panels showing the task design across all figures, and kept consistent across all panels. We have also added more panels to further break down each experimental task design throughout the manuscript.

Figure 5f: though it is statistically significant, 8 out of 17 subjects showed increased (or unchanged) RT as opposed to reduced RT.

We also noted this trend, which we believe is due to a floor effect. We can see from the absolute reaction times in panel g that the participants whose reaction times are already fast in the non-rewarded condition show generally less change in reaction times with reward.

Thanks to the reviewer’s comment, we also noted an important mistake in the analysis of our proprioception-cued reaction times. In the main text and in our code, we define reaction times by finding when the triceps EMG signal rises 5 times above the trial baseline standard deviation for 5 ms consecutively. However, this is a very stringent criterion, which leads to a significant number of trials being thrown out for not meeting it (~20% of all trials across participants). In the updated version of the manuscript, we now use a more typical 3 standard deviations, which leads 91 out of 1836 trials being removed (~4.9%). Critically, this does not change the results in any meaningful way: we still observe a significant decrease in reaction times with reward, a widespread increase in feedback gains across all recorded muscles, and about a third of participants showing little to no change in reaction times with reward, as per the reviewer’s initial comment (see updated manuscript).

Figure 6: Is the MT improvement a result of a movement speed increase not related to the cursor jump response that happens during the movement?

That is correct. Each of the tasks serve to highlight one aspect of movement relating to a feedback response, but it does not mean that the other feedback responses are unchanged (or for that matters even the feedforward drive). Therefore, if any other aspect of movement was sensitive to the rewarding context, the movement times would still benefit from those, even if this is not picked up by the particular task design. Assessing movement times is useful as a control to ensure that the varying reward was indeed effective at motivating participants in the first place. Otherwise, one may (rightfully) ask whether the varying reward was effective at all when faced with a non-significant result for a change in latency or in feedback gain.

The target jump condition is theorized with longer latency than the cursor jump condition (Figure 8). Is this really the case? It appears that their RTs are similar.

In this study they appear similar indeed, which diverges from the literature. For that reason, we have indicated in figure 1 captions that “*Latencies indicated here reflect the fastest reported values from the literature and not necessarily what was observed in this study.*”

The paper proposes to classify feedback control by sensory domain and response latency, not by function. The argument is that "…it does not macth any observed pattern here (Figure8)". But what pattern does this refer to? The fact that response latency and modality matter for the "reward effect" does not justify ditching the use of "function." In my opinion, the more apparent pitfall would be the loose use of "function" terms for different tasks. For instance, I wonder whether associating the target jump task with online tracking of the goal is reasonable. Tracking is typically referred to as using an effector to follow or spatially match a moving visual target. It is certainly not the case for a reaching movement to a possibly-changing target that has not been attained yet. It appears to me that for the same function, people can design drastically different tasks; that's the real problem that the current study should emphasize.

We thank the reviewer for this important point. We agree with this, and it aligns with what we are arguing for, in that categorization based on sensory modality or neural pathway are factual and therefore objective, making it a more normative framework. However, categorization by function is also useful in some contexts, which we emphasize with this underlined segment (section 3.3):

“Therefore, while it may have value at a higher-order level of interpretation, we argue that a categorization of feedback loops based on function may not always be the most appropriate means of characterizing feedback control loops.”

We appreciate that the manuscript will greatly benefit from making the reviewer’s point more explicit. We have edited the end of section 3.3 to read:

“This may partially stem from the inherent arbitrariness of defining function and assigning a specific task to that function. In contrast, categorization based on neural pathways, neural regions involved, and sensory modality may result in more insightful interpretations, because they are biologically grounded, and therefore objective means of categorization.”

Reviewer #2 (Recommendations for the authors):

The question on how reward or value impacts feedback processes is important to understand. Previous studies highlight how reward impacts motor function. Given feedback is an important aspect of motor control, it is useful to know which feedback responses may be specifically impacted or altered by reward.

The manuscript uses a functional taxonomy suggested by Scott (2016) to define the behavioural contexts examined in the paper. A clear strength of the manuscript is the systematic evaluation of these feedback processes with distinct latencies. This resubmission addresses several issues raised in the initial review. Notably, most experiments have been redone to better align with the defined behavioural processes and the use of more standardized experimental approach and analyses techniques across experiments.

There are some methodological issues that are either poorly described or seem to be a problem. From the methods section, it looks like only the R2 and R3 epochs (50 to 100ms) were examined for each experiment. This doesn't make sense for experiments such as target and cursor jumps that only lead to EMG responses at ~100ms after the disturbance.

We agree that focusing exclusively on the R2 and R3 epoch would be an issue for the reason mentioned above. The method section 4.6, second paragraph defines the time window in which the gains were estimated to be a 50 ms window starting at the latency time:

“To compute feedback gains, for each feedback response considered we defined a 50 ms window that started at that response’s latency found for each participant independently using ROC analysis.”

As noted by the reviewer, since the target jump and cursor jump conditions start at ~100 ms, then the window boundary will start at that point as well, and finish 50 ms later. To make this methodological point clearer, we have added the following at the end of section 2.5:

“Consistent with the previous experiments, we assessed feedback gains on all five recorded muscles in a time window of 50 ms following each participant’s response latency for the experiment considered (here a cursor jump).”

All figures throughout the manuscript also now indicate in the caption for the feedback logratio panel:

“Log-ratio G of feedback gains in the rewarded versus non-rewarded conditions in a [relevant duration here] ms window following the [relevant feedback response here] onset.”

As well, magnitude changes are monitored for 4 different muscles, but why is there only one latency examined (last panels in most figures) and which muscle is being used is not clear for each experiment.

EMGs were recorded for the same 5 different muscles for all experiments. The first paragraph of section 2 states:

“We recorded electromyographic signals (EMG) using surface electrodes placed over the brachioradialis, triceps lateralis, pectoralis major (clavicular head), posterior deltoid, and biceps brachii (short head).”

Additionally, each time the feedback gains are assessed throughout the manuscript, each muscle name is repeated with the associated statistics, e.g.:

“Feedback gains were greater in the rewarded condition for the triceps, deltoid, and brachioradialis in a 50 ms window following LLR onset (biceps: W=98, r=0.72, p=0.12; triceps: W=136, r=1, p=4.4e-4; deltoid: W=135, r=0.99, p=5.3e-4; pectoralis: W=96, r=0.71, p=0.15; brachioradialis: W=129, r=0.95, p=1.6e-3; Figure 3f-h).”

The same five muscles are used across all experiments where EMG signals are recorded, which was one of the main requests of the previous round of reviews. For each figure throughout the manuscript, the same five muscles are also enumerated in the feedback gains panel.

To make this clearer, in the methods section 4.5, the first sentence now reads:

“For each experiment, the EMG signals of the brachioradialis, triceps lateralis, pectoralis major (clavicular head), posterior deltoid, and biceps brachii (short head) were sampled at 1000 Hz, band-pass filtered between 20 Hz and 250 Hz, and then full wave rectified.”

I think some of the points raised in the discussion need to be developed more including the addition of pertinent literature. Specifically, the section on 'categorizing feedback control loops', brings up the point that it might be better to not use functional processes as a framework for exploring feedback control. Instead, they suggest categorization should be based on neural pathways, neural regions and sensory modalities. There are no citations in this section. However, in the conclusion it appears they suggest this paragraph is about using a bottom-up approach based on pathways and neural regions rather than a top-down functional approach. If that is their message, then the bottom-up approach has been around since Sherrington (see also recent ideas by G. Loeb) and so it would be worthwhile to include some integration of existing ideas from the literature (if they are related). While this is a worthwhile conversation, I think the authors should be cautious in concluding from this one behavioural study on reward that we should just ignore functional processes. Perhaps the problem is the term of linking complex functional processes to single 'feedback loops' as such processes likely engage many neural pathways Notably, the present discussion states that the cortico-cerebellar feedback loop was not considered in the present study. However, it likely was involved. In fact, in the 1970s the R3 response was commonly associated with the cerebellar-cortical feedback pathway. The richness of brain circuits engaged after 100ms is likely substantive. Thus, there needs to be some caution on linking these behavoural experiments to underlying brain circuits. The value of thinking about behavioural function is not because function can be found in a single brain region or pathway. Rather it is to ensure tasks/experiments are well defined, providing a sound basis to look at the underlying circuits and neural regions involved.

We thank the reviewer for the points brought up here as they enrich the content of the discussion on that matter. We have already developed this section in response to reviewer #1, and have added the following at the end of section 3.3 to further expand with reviewer #2’s comments:

“More generally, our results provide additional evidence in favour of a bottom-up approach to understanding the brain as opposed to a top-down approach. This approach is described as early as Sherrington (Burke, 2007; Sherrington, 1906), who put forward an organizational principle of the central nervous system tied to sensory receptor properties (extero-, proprio-, intero-ceptors, distance-receptors). More recently, Loeb (2012) proposed that the existence of an optimal high-order, engineering-like control design in the central nervous system is unlikely due to the constraints of biological organisms, a theory further detailed by Cisek (2019) from an evolutionary perspective.”

Following reviewer #1’s comments, we have also toned down the language of this section to ensure it does not represent a rebuttal greater than this study alone warrants.

From above, the key points I think need to be considered are defining the time epochs under study for each experiment (need to ensure reader knows for each experiment) and why latency in only one muscle and which one for each study. The other point is to expand section on categorizing feedback loops with the existing literature, as suggested above.

From the points raised above we do appreciate that the manuscript would greatly benefit from the methodology being more clearly described at the beginning of the Results section. We have added two paragraphs in section 2.1 for that purpose (see main text). We have also added panels describing the experimental design at hand for each figure across the manuscript.

The diagrams are very well organized. However, I wonder if it would be useful to show the hand speed against time to highlight your point that movement times were faster in rewarded trials in either Figure 1 or 2. This may not be necessary for all figures, but the first few to give the reader some sense of how much hand speed/movement time was altered.

We thank the reviewer for this suggestion. This has now been done throughout the manuscript.

Reviewer #3 (Recommendations for the authors):

It is known that if one can obtain a reward, motor performance improves. The authors' aim is to answer the question which of the nested sensorimotor feedback loops that underly motor performance is/are affected by expected reward (and how).

The authors provide a large set of experimental conditions and show that their manipulation of the reward affects some aspects of the response to the perturbations in a latency-dependent way. The experiments are designed very similarly, so easy to compare. The authors succeed to a large extent in showing very convincingly that reward affects some feedback loops, but not others. However, there are some weaknesses, mainly in how the authors deal with the possibility that latencies might depend on reward. If this is the case, then the analysis becomes problematic, as the way the gain ratio is defined (based on differences) assumes equal latencies. The authors do not have a solid method to disentangle effects on latency from effects on gain.

We thank the reviewer for bringing up this point. We acknowledge that there are advantages and disadvantages with either analysis methods. The reviewer’s last sentence summarizes this situation well: “The authors do not have a solid method to disentangle effects on latency from effects on gain”. Therefore, ideally one would observe the same result with each analysis method.

In Author response image 2, we show the feedback gain log-ratios for fixed time windows. The SLR time window has a 25 ms width and the other time windows have a 50 ms width, similar to the original analysis from the main text. The time windows are fixed: the SLR time window starts at 25 ms post-perturbation, the LLR time window starts at 50 ms, the target selection time window starts at 75 ms, and the remaining time windows (target and cursor jump, and reaction times) start at 100 ms. Overall, we observe a similar pattern of increase in feedback gains as with the original, latency-locked estimate of feedback gain log ratios (Figure 9).

**Author response image 2.**

A weakness is that there is no clear theory to identify feedback loops. The most evident example is the use of the functions (the colour code in Figure 1). For instance, what is the difference between 'goal-switching' and 'on-line action selection'? To me, these two refer to the same function. Indeed, the latencies for on-line goal switching depend on the design of the experiment, and even be as short as those for on-line tracking of the goal (Brenner and Smeets 2022). Also, the difference in labeling the SLR and LLR is not straightforward. In figure 2, it is clear that there is a LL reflex that depends on reward, the function here is on-line control of the limb.

We agree that the use of function-based categorization inherently carries arbitrariness. In this study it is included for scholarly completeness, but in the discussion, we argue against it as well for similar reason (section 3.3). This also relates to other reviewers’ points on the matter. Based on this comment and the other reviewers’, we have expanded this section to better communicate the points developed here (see main text).

In the experiment of figure 3, that also yields a LLR, I see no reason why the function would not be the same, despite the task being different. The splitting of the LLR in a R2 and R3 makes things even more complicated.

Indeed, the LLR arises essentially in all experiments that employ a mechanical perturbation. For instance, besides figure 2 and 3, figure 4 shows a LLR response (figure 4 panel d), even though it is a different experimental design. The main point of the experiment and contrast used in figure 3 is that it leads to a divergence in EMG signals for the triceps muscle exactly when the LLR should arise (Pruszynski et al., 2008, Rapid Motor Responses Are Appropriately Tuned to the Metrics of a Visuospatial Task). This allows us to compute a clear estimate of the LLR latency for each individual and in the rewarded and non-rewarded conditions independently in that experiment.

The task in figure 3 is the same task as in figure 2, but the contrast used is different (see figures 2d and 3c). Based on the reviewer’s comment, we appreciate that this methodological approach was not clear enough at the beginning of the results, and we added two paragraphs to amend this in section 2.1 (see main text), as well as more panels to all figures breaking down the experimental design and contrast at hand.

We have also removed the R2-R3 comparison, as we agree in its current form, the manuscript does not clearly benefit from this comparison and detracts from the main motivation.

Lastly, it is interesting that the authors label the feedback loops involved in experiment 3 to differ from those in experiment 2, although they have the same latency and same sensory input.

The latencies observed for figure 2 are in the 20-35 ms range, and those of figure 3 are in the 40-75 ms range (last panel for each figure).

A second weakness is the discussion on the latency of the responses. We have shown previously that conclusions about effects of a manipulation on latency depend critically on the way latency is determined (Brenner and Smeets 2019). So the effect of reward on latency might be an artifact, and should be confirmed by using other methods to determine latency.

We thank the reviewer for pointing out this study. It is a particularly interesting work in that it assesses several different means of estimating latencies. This study tests each method on empirical data, for which the “true” latency is unknown. In addition, testing the same methods on simulated data, for which the true latency is known, would naturally expend on these results, enabling us to assess each method’s actual accuracy, systematic bias, and robustness to noise. It would also allow us to compare these alternative methods to the method we use (Receiver Operating Characteristic; ROC).

To assess the true bias and robustness of each alternative method from Brenner and Smeets (2019), we created simulated data that diverge according to a piecewise linear function (Author response image 3).

**Author response image 3.** Simulated data with gaussian noise (noise standard deviation = 0). 2).

We then tested each reaction time estimation method’s accuracy as we varied noise, which was a random gaussian process centred on 0. As indicated above, an advantage of this approach is that the ground truth is known, enabling meaningful normative comparisons.

We can see from Author response image 4 that the extrapolation method, which was found to be the most reliable in Brenner and Smeets (2019) performs particularly well with low-noise data like the data used in the original study, which were kinematic data. However, for data exhibiting higher noise, we observe that ROC analysis (coupled with segmented linear regression, as in our current study) performs best.

**Author response image 4.** Estimated latency as noise standard deviation in the simulated data was varied. The blue line indicates the true latency of signal divergence.

This leads one to ask where an EMG signal such as the one we have in our study would be located on the noise axis of Author response image 4. In Author response images 5 and 6 we show the extrapolation lines from the extrapolation method, fitted on the difference (divergence) of mean EMG for the flexion and extension jump conditions of the cursor jump task (Author response image 5). We fitted the extrapolation line on rewarded and non-rewarded conditions independently (green and red, respectively). We can see that the resulting latencies (intersection of the extrapolation line and the null line, in black) occasionally represent an unreliable estimate. In comparison, the segmented linear fits on the area-under-curve (AUC) data are more reliable (Author response 6). This suggests that the EMG data we are dealing with in this study more closely relates to the high-noise regime from the simulation. The same general result was observed for other experiments in our study. We believe that this is mainly tied to the inherently noisy nature of EMG signals compared to e.g., kinematic data rather than the specifics of a given task design.

**Author response image 5.** Extrapolation method from Brenner and Smeets, 2019, on triceps EMG from the cursor jump task.

**Author response image 6.** ROC method from Weiler et al. , 2015, on triceps EMG from the cursor jump task. The solid blue lines indicate the segmented linear fits, and the “knee” of this fit is highlighted with vertical dashed lines.

The authors argue in their rebuttal against using fixed time-windows. I am not convinced for 3 reasons: 1) by using a data-driven definition of the various reflex-epochs, the authors compare responses at different moments after the perturbation. We see for instance in figure 2h that the latency obtained for a single participant can differ 20 ms between the rewarded and non-rewarded condition (without any meaning, as the two conditions have the same latency, and the length of the arm was also not changed), so that the gain compares different epochs without any reason. Thus any uncertainty in the determined latency affects the values obtained for the gain-ratio. 2) the paper that defined these epochs (Pruszynski et al. 2008) used fixed periods for R1, R2 and R3. 3) the much older monkey-work by Tatton et al. reported consistent latencies for R1 and R2, and only variable latencies for R3. The authors do the opposite: assume a fixed latency of R3 (relative to R20, and variable for R1 and R2.

We thank the reviewer for bringing up this point. As mentioned in the response to the reviewer’s first comment, using fixed latencies for computing feedback gains yields the same result as the method employed in the main text.

We acknowledge that the use of the R1-R2-R3 terminology is confusing here as it does not match the canonical definition. For this reason, and because this analysis detracts from the main points of the manuscript in its current form, we have removed this analysis from the study.

A third weakness is that the authors seem to claim that the changes in the feedback are causing better performance. The first aspect that troubles me is that only one factor of performance is provided (speed), but higher speed generally comes at the cost of reduced precision, which is not reported.

Indeed, there is a speed-accuracy trade-off in motor control (Wickelgren, 1977). However, rewarding participants tends to break that speed-accuracy trade-off, in that movements can be faster and as accurate (during upper arm reaching movements; Codol et al. 2020, J Neurosci) or faster and more accurate (during eye saccades; Mahonar et al. 2015, Current Biology). In reaching movements, one mechanism that explains this phenomenon is an increase in endpoint stiffness in a rewarding context at the end of the movement (Codol et al. 2020, J Neurosci). Importantly, the same study shows this increase in stiffness is absent at movement onset, which would have been an important confound in the current study otherwise (that last point was also discussed in the previous round of reviews).

DOIs for references

Wickelgren (1977): 10.1016/0001-6918(77)90012-9

Codol et al. (2020): 10.1523/JNEUROSCI.2646-19.2020

Manohar et al. (2015): 10.1016/j.cub.2015.05.038

By the way, MT (i.e. end of movement) is not defined in the paper.

We thank the reviewer for pointing out this missing information. We have added in section 4.6:

“Movement times were defined as the time between the occurrence of the mechanical perturbation being applied and entering the end target in the proprioceptive tasks (In-Out task, Target Selection task and Proprioception-cued Reaction Time task). They were defined as the time between leaving the end position and being in the end target with a radial velocity of less than 10 cm/sec in the visual tasks.”

The second aspect is that I think they are not able to determine causality using the present design. The authors do not even try to show that feedback and MT are correlated. The authors should then limit their claim to their finding that reward changes movement time and feedback mechanisms.

MTs are provided here as a control measurement to ensure rewarding context impacts performance at all. This is a suitable control measurement because MTs becoming shorter with reward is a well-established phenomenon. But performance improving with reward is generally not constrained to shorter MTs, and this is well-documented as well. This study does not attempt to characterize all the ways behaviour is improving because it is already done. Rather, we ask through what means may the sensorimotor system enable those behavioural improvements, specifically targeting the feedback control system. Some feedback loops will contribute to these improvements, and some will not, but all feedback loops (and other mechanisms such as stiffness control and noise reduction) are still taking place in every experiment presented here. For instance, we can see a clear LLR response in the triceps EMG of the Target Selection task (figure 4). Nevertheless, each experiment is designed to make a specific feedback response easily observable and so quantifiable, which then allows us to unambiguously assess whether that feedback response has changed with reward. In all experiments presented here, we should expect a change in MTs with reward, signifying that rewarding context is integrated by the sensorimotor system into the control policy. But we will not always see a correlation with the specific feedback response that task is probing, because that specific feedback response may not integrate that rewarding context. If so, the change in MT would be enabled by changes in other mechanisms that this specific task is not probing but that is still contributing to behaviour. So, correlation between feedback-related variables and MTs is not always expected and is in itself uninformative.

A fourth weakness is their flexibility in the choice of their dependent measures, and (related) the excessive use of hypothesis testing (p-values). For instance, they measure the EMG from five muscles, and use sometimes all signals, and sometimes restrict themselves to the ones that seem most suited (e.g. when claiming that the latency is significantly reduced). Not analysing some signals because they are noisier gives an impression of p-hacking to me. Furthermore, by using more than one signal to test a hypothesis about a feedback loop, they should use a (Bonferroni?) correction for multiple testing. By reporting p-values rather than the differences themselves, the authors obscure the sign of the difference. A better strategy would be to report all differences with their confidence intervals and base your conclusion on this (the reader can check to what extent this ensemble of results indeed supports your conclusion).

In the context of ROC, “noisy” does not refer to the standard deviation of EMG signals, but rather the noise in the classification signal (area under curve, AUC) to produce above-chance classification. Even with similar standard deviation of the EMG, if a muscle’s activity does not diverge between conditions, one cannot estimate the latency of that (non-existent) divergence. For our study, this situation would indicate that there is no relation between the geometry of the task (orientation of movement) and the geometry of the muscle (orientation of pull) considered. Since there is little redundancy in the geometry of the muscles we recorded from (and in the musculoskeletal system overall), it is essentially impossible to find an experimental design that would result in two muscles diverging for the same movement, forcing one to decide pre-collection which muscle to emphasize. Here, we decided to emphasize the triceps in all our experiments to remain consistent with the experiment measuring the SLR and LLR, since this is the experiment that the reviewers had found most convincing and appealing in the first round of review.

We could have designed the experiments to emphasize another muscle (i.e., which muscle we want to emphasize is arbitrary). But having only one muscle whose EMG signal diverges between conditions prevents us from exploring all the muscle signals post-hoc and picking whichever would be the most convenient. In other words, since the muscle to analyse must be declared pre-collection, one cannot decide on post-hoc changes once data is collected. That situation is in fact robust to p-hacking rather than prone to it.

In addition, since we use the same muscle across experiment for estimating latencies, there is no rationale for changing post-hoc the muscle considered for one experiment without having to change it for all five experiments. And since the first experiment (assessing the SLR and LLR) is preserved from the first version of this draft, this acts as an anchor point preventing unchecked data mining with respect to which muscle to use for latency estimation.

More generally, we would like to point out that by re-designing and re-collecting four out of five experiments, the original (first) draft of this study acts as a check against data mining. The scientific question at hand, the variables of interest (feedback gain and latency), the analysis to estimate them (difference of integrals over a time window, ROC analysis), and statistical tests used (Mann-Whitney rank-sum tests) are all strictly the same as in the first draft.

The individual data of each statistical test is shown in the figures, alongside the group mean and bootstrapped 95% confidence intervals.

The authors might want to add information on the correlation between changes in feedback gain/latency and changes in MT.

We thank the reviewer for this suggestion. We discuss this point in the responses above.

P2 "More recent studies outline" The studies that follow aere not more recent tghan the ones in the previous paragraph

We thank the reviewer for bringing up this point. This sentence now starts with “Some studies outline”.

Figure 2b: explain somewhere how the trajectories are averaged. As the response latencies might vary from trial-to-trial, averaging might introduce artifacts. Explain the method, and indicate in the bottom half of the plot which of the 15 curves belongs to the participant shown in the upper half.

The kinematics shown in panel c (and equivalent panels throughout the manuscript) are to provide a sense of how a trial movement looks to the reader. For clarity, these averaged kinematic data were not used to calculate latencies or feedback gains, or for any other analysis, as the reviewer may already be aware.

To compute the kinematics averages, the cartesian position over time was interpolated between the end of the trial baseline and the moment that the cursor was in the end target with a velocity of less than 10 cm/sec. We then averaged across those 100 interpolated samples.

We appreciate that averaging kinematics over the whole trial might introduce artefacts. Accordingly, we removed the averaged trajectories from the figures and main text since we do not make use of these averages and since trial-by-trial trajectories are sufficient to provide a sense of how a trial unfolds over time to the reader as was our primary purpose.

Figures 2d,e, 3d,e, etc: Unclear why the left panels with the trial-baseline are included, as it is visible in the right panels as well (from -50 to 0). In the right panel, use the same x-axis, so responses are more easily comparable. Please indicate the time-window under study by a bar on the time-axis. I understand that the time-window used varies a bit from participant to participant, you might show this by letting for instance the thickness or saturation of a bar at each time indicate the number of participants that contributes to that part. Also: use milliseconds to report the difference in MT.

We thank the reviewer for these suggestions. The x-axis of the right and left axes of EMG panels are now scaled identically to allow for better comparisons.

The period from -50 to 0 in the right axes are pre-perturbation EMGs, which are different from the trial baseline signals shown in the left axes. As noted in the method section 4.5 (“EMG signal processing”):

“For all experimental designs, trial baseline EMG activity was measured in a 50 ms window from 350 to 300 ms before displacement onset, while participants were at rest, background loads were applied, and after targets and reward context were provided. For the Target Jump and Cursor Jump tasks, this was measured in a 50 ms window from 350 to 300 ms before target appearance instead of before displacement onset because movements were self-initiated, and displacements occurred during the movement.”

From the above quote, we can see that the 50 ms preceding the perturbation are not the same as the trial baseline for any of the experiments. For clarity, we have also added in section 4.5:

“For all experiments, the average trial baseline EMGs are displayed on a left axis next to the axis showing perturbation-aligned EMG signals.”

The left and right axes both contain the same amount of participant through the entire period shown (specifically, all of them).

We have now changed the units of δ MT panels from sec to ms throughout the manuscript.

Figure 2f: The caption text "Feedback gains following SLR onset" is not informative and even wrong. It is a ratio, and it is from a limited time-widow.

This caption now reads “Log-ratio *G* of feedback gains in the rewarded versus non-rewarded conditions in a 25 ms window following SLR onset”. We have also carried this change throughout in the other figures for the equivalent panels, changing to “*50 ms window*” where appropriate. The “*Log-ratio G”* is also now formally defined in panel 2i.

Statistical reports in the text makes reading hard (e.g. on page 5 21 times an "="). Try to move these numbers to the figures or a table.

Make sure that you use similar phrases for similar messages. E.g., the analysis of MT in 2.1 is described totally different from that in 2.2, whereas the analysis is the same. In a similar fashion, don't use "Baseline EMG" for two different measures (the one based on 4 separate trials, and the one based on al trials).

We thank the reviewer for pointing this out. The description of MT analysis in section 2.1 is now closer to the description in section 2.2.

We now refer to the baseline EMG activity before each trial as “trial baseline”, and specify it in the methods section throughout. We also do not refer to the EMG signals recorded during the 4 trials before the task as baseline anymore. Instead, we refer to the resulting value as the “normalization scalar”.

P7: The authors report separate values for the gain-ration for the R2 and R3 epochs, but don't show these, bit only a single combined ratio.

We thank the reviewer for bringing up this point. The R2-R3 comparison is now removed from the manuscript, as detailed in the above responses.

P8, figure 3d (lower): how is it possible that we see that the green curve responds clearly earlier than the purple, but we do not see this in figure 3i?

The difference is not large enough and consistent enough across participants to yield a significant result. The discussion above provides a more detailed discussion on the sensitivity of the estimation technique used to produce panel i, which is an ROC analysis. We also recommend the methods section of Weiler et al. (2015, referenced in main text throughout) for a thoughtful and in-depth analysis of the sensitivity of ROC analysis to noise.

P9 (figure 4): I am puzzled by the relation between panel e and f. Panel e looks very similar to the corresponding panel in figure 3 (green clearly different from purple), but the sign of the gain log-ratio is opposite to that in figure 3.

If this point refers to the fact that Figure 4’s log-ratios are non-significant while the EMGs in panel h show a stronger response in the rewarded (green) condition than the non-rewarded (purple) condition, this is because the difference arises later than the time window during which the feedback loop that we are assessing takes place (see figure 2i, the grey area stops eventually).

It is confusing to redefine the concepts 'R1', 'R2", and 'R3'; in the present paper these refer to variable intervals that depend on the participant, whereas the paper that defined these intervals (Pruszynski et al. 2008) used fixed intervals.

We now appreciate that this may be confusing, and have removed this terminology from the main text, as mentioned above. We thank the reviewer for bringing up this concern.

P26 "we defined a 50 ms window" What is defined as 50ms? The R1 is only 25, and the complete set R1-R3 is 75 ms.

We appreciate from the all the reviewers’ comments that the R1-R3 terminology is confusing, so we removed it from the manuscript. What is defined here is a window, that is neither R1, R2, R3 nor any combination of these, and whose width is 50 ms.

P28 De reference to De Comité et al. 2021 is incomplete. I guess you want to cite the 2022 eneuro paper.

We thank the reviewer for pointing this out. This is now amended.

P32 De reference to Therrien et al. 2018 is incomplete. I guess you want to cite their eneuro paper.

We thank the reviewer for pointing this out. This is now amended.
